# DNA supercoiling enhances DNA condensation by ParB proteins

**DOI:** 10.1093/nar/gkae936

**Published:** 2024-10-23

**Authors:** Alejandro Martin-Gonzalez, Miloš Tišma, Brian T Analikwu, Anders Barth, Richard Janissen, Hammam Antar, Gianluca Kemps, Stephan Gruber, Cees Dekker

**Affiliations:** Department of Bionanoscience, Kavli Institute of Nanoscience Delft, Delft University of Technology, Van der Massweg 9, 2629HZ Delft, Netherlands; Department of Bionanoscience, Kavli Institute of Nanoscience Delft, Delft University of Technology, Van der Massweg 9, 2629HZ Delft, Netherlands; Department of Bionanoscience, Kavli Institute of Nanoscience Delft, Delft University of Technology, Van der Massweg 9, 2629HZ Delft, Netherlands; Department of Bionanoscience, Kavli Institute of Nanoscience Delft, Delft University of Technology, Van der Massweg 9, 2629HZ Delft, Netherlands; Department of Bionanoscience, Kavli Institute of Nanoscience Delft, Delft University of Technology, Van der Massweg 9, 2629HZ Delft, Netherlands; BITZ Transformation Lab, Deggendorf Institute of Technology, 94363 Oberschneiding, Germany; Department of Fundamental Microbiology (DMF), Faculty of Biology and Medicine (FBM), University of Lausanne (UNIL); CH-1015 Lausanne, Switzerland; Department of Bionanoscience, Kavli Institute of Nanoscience Delft, Delft University of Technology, Van der Massweg 9, 2629HZ Delft, Netherlands; Department of Fundamental Microbiology (DMF), Faculty of Biology and Medicine (FBM), University of Lausanne (UNIL); CH-1015 Lausanne, Switzerland; Department of Bionanoscience, Kavli Institute of Nanoscience Delft, Delft University of Technology, Van der Massweg 9, 2629HZ Delft, Netherlands

## Abstract

The ParABS system plays a critical role in bacterial chromosome segregation. The key component of this system, ParB, loads and spreads along DNA to form a local protein–DNA condensate known as a partition complex. As bacterial chromosomes are heavily supercoiled due to the continuous action of RNA polymerases, topoisomerases and nucleoid-associated proteins, it is important to study the impact of DNA supercoiling on the ParB–DNA partition complex formation. Here, we use an *in-vitro* single-molecule assay to visualize ParB on supercoiled DNA. Unlike most DNA-binding proteins, individual ParB proteins are found to not pin plectonemes on supercoiled DNA, but freely diffuse along supercoiled DNA. We find that DNA supercoiling enhances ParB–DNA condensation, which initiates at lower ParB concentrations than on DNA that is torsionally relaxed. ParB proteins induce a DNA–protein condensate that strikingly absorbs all supercoiling writhe. Our findings provide mechanistic insights that have important implications for our understanding of bacterial chromosome organization and segregation.

## Graphical Abstract

**Graphical Abstract:**
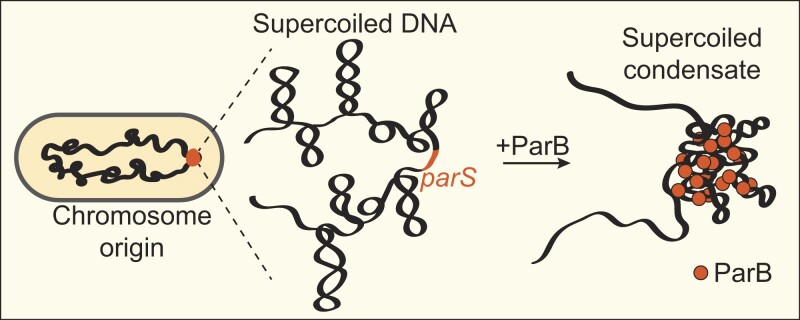

## Introduction

**Figure 1.**
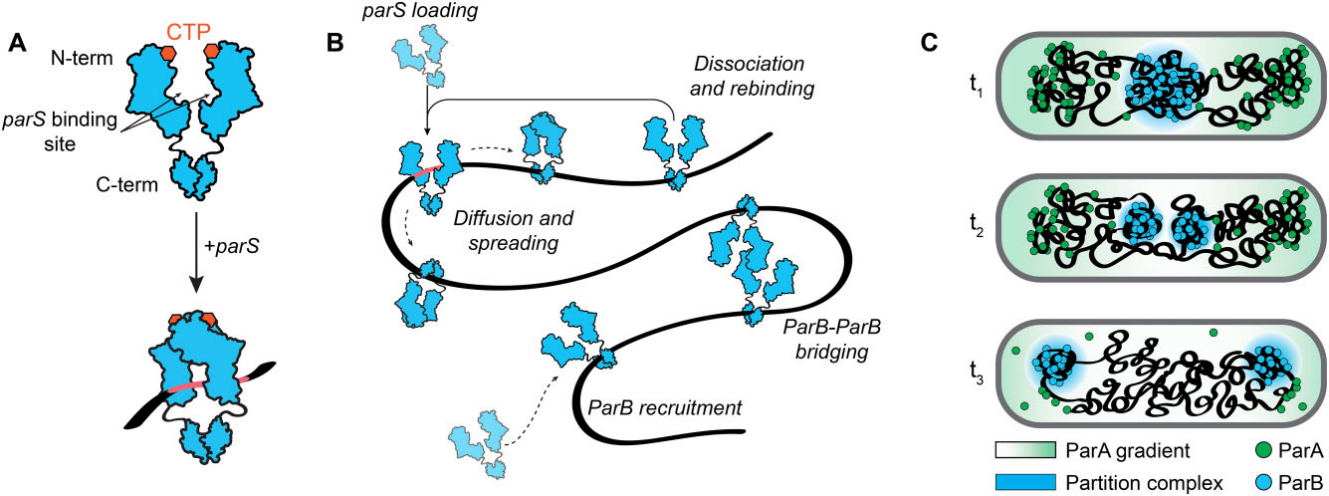
ParB binding to DNA and partition complex formation. (**A**) Schematic representation of ParB dimer structure and loading to DNA*_parS_*. Monomer units of ParB each bind a CTP nucleotide at their N-terminal domain (6). Upon recognition of parS site, ParB dimer undergoes a conformation change and forms a topological clamp around the DNA (11–13). (**B**) ParB loading, spreading and bridging on the DNA. Upon loading to the *parS* site (top left), ParB dimers lose the affinity to *parS* and start diffusing along the DNA (6,10). Upon CTP hydrolysis, ParB proteins either (i) dissociate from the DNA and recycle to *parS* (11), (ii) recruit a new ParB protein onto the DNA from the surrounding (44) or (iii) form a dynamic ParB–ParB bridge with another DNA-bound ParB dimer – which is essential for partition complex formation (14,17,24,41). (**C**) Schematic depicting directional movement of the partition complex in chromosome segregation. A large ParB partition complex is formed right after origin replication which contains nearly all cellular ParB proteins. The partition complex recruits SMC complexes (not shown in the schematic) that help to split two nascent origins (27,28). Nascent partition complexes interact with the nearby ParA molecules and start directionally moving towards the bacterial cell pole of the cell via diffusion ratchet mechanism (29–31).

Reliable segregation of chromosomes to daughter cells is a fundamental requirement for the stable propagation of all living organisms. The ParABS system is the primary mechanism responsible for the faithful segregation of chromosomes in the majority of bacteria (1,2). It is comprised of an ATP-hydrolase partition protein A (ParA) (3,4), a CTP-hydrolase partition protein B (ParB) (5,6) and a 16-base pair centromeric sequence known as *parS* that is present in multiple copies near the origin of replication (7). While ParA proteins bind non-specifically to the DNA to cover the entire chromosome (8,9), ParB proteins specifically load onto DNA at the *parS* sequence (6,10,11). Prior to this binding, two monomers of ParB protein form an open dimer via their C-terminus, exposing the DNA-binding domain for the *parS* site recognition (6,11) (Figure 1A). The N-terminal domain of ParB can bind CTP nucleotide, which is a crucial regulator of ParB activity (6,10–13). Upon *parS* binding, a ParB dimer changes the conformation to a closed state (‘clamp’) around the DNA molecule while it sandwiches two previously bound CTP molecules between two N-termini (11,12). After forming a clamp, the ParB dimer loses the affinity to the *parS* site and diffuses laterally along the DNA spreading to distances of up to 10 kb (Figure 1B) (11). Eventual hydrolysis of both CTP molecules, after ∼1–2 min, destabilizes the ParB clamp and the monomers revert back to an open state (11,12). This open clamp can now either dissociate from the DNA molecule or connect to a nearby ParB dimer forming a dynamic protein-protein bridge (14–17). Dissociation ensures that ParB proteins recycle and reload to the *parS* site ensuring the maintenance of a high local ParB concentration near the origin, while the dynamic bridging is important for the formation of a higher-order nucleoprotein structure known as a partition complex (18–20). There are multiple models for the partition complex formation, such as nucleation and caging between ParB and DNA (20,21), liquid–liquid phase separation (22,23) or bridging-induced condensation (14–18,24,25), but they all rely on the presence of DNA-bound ParB proteins that exhibit self-self interaction and the bridging of distal segments. Formation of the partition complex near the origin of replication further promotes the loading of SMC proteins (Structural Maintenance of Chromosomes) (26–28) and interacts with a ParA gradient along the nucleoid (Figure 1C) (23,29–31), which induces a directional movement of the nascent origin to the daughter cell.

In cells, transcription exerts prominent forces and twists on the genomic DNA (32,33). An RNA polymerase transcribing a gene will continuously create positive supercoiling downstream and negative supercoiling upstream of the transcription site (34–37). When such torsional tension is built up, the DNA will twist and form plectonemes that are extended intertwined DNA helices (37–39). Many other cellular processes modulate and regulate the supercoiling within the genome through actions of topoisomerases and nucleoid-associated proteins. Vice versa, supercoiling is known to affect many processes in bacterial cells by changing the protein-binding affinities and spatial organization of the DNA (32,40). Recent *in-silico* studies (41,42) suggested that supercoiling may, for example, strongly change the dynamics of the ParB–DNA partition complex by modulating the interactions between distal segments of the DNA.

Here, we examine the effects of DNA supercoiling on ParB diffusion and condensation dynamics using a single-molecule visualization assay (43). We measured the diffusion of single ParB dimers on supercoiled and non-coiled DNA molecules, and found that the presence of supercoiling reduces the residence time of ParB on the DNA. Furthermore, we measured the degree of ParB–DNA condensation induced by ParB proteins on supercoiled DNA as well as the changes in plectoneme dynamics and localization in the presence of ParB. We observed that all plectonemic structures were absorbed within the ParB–DNA condensate. Lateral DNA flow and atomic force microscopy (AFM) confirmed that supercoiled DNA in the presence of ParB yielded a collapsed structure that absorbed all supercoiling writhe. This experimental study reveals the interplay of DNA supercoiling and ParB–DNA condensation, two fundamental and essential processes that govern bacterial genome organization and segregation.

## Materials and methods

### ParB purification and fluorescent labeling

The expression constructs were prepared using pET-28a(+) plasmid backbone with the inserted *Bacillus subtilis* ParB^L5C^ gene in it [originally published in (16) and modified by Soh et al.(6)]. In brief, ParB was amplified from the genome using primers STM682 and STM683, which introduce BamHI and NcoI restriction sites into the polymerase chain reaction (PCR) product (Table 1). Additionally, the primer STM683 introduces a mutation at the Lys5 in the *parB* gene and converts it to a Cys, which is later used for protein labeling via click chemistry reaction with maleimide dyes. The final plasmid was constructed using a standard NEB protocol for BamHI and NcoI digestion, which was used both for the ParB^L5C^ PCR product and pET-28a(+) plasmid (Novagene), followed by a T4 DNA ligase (New England Biolabs, M0202L) ligation for 15 min at room temperature prior to transformation to chemically competent BL21-Gold cells. We expressed recombinant proteins in *E. coli* BL21-Gold (DE3) for 24 h in ZYM-5052 autoinduction medium at 24°C. Purification of ParB^L5C^ variant, used for fluorescent labeling, was performed as described before (6,44). Briefly, we pelleted the cells by centrifugation and subjected them to lysis by sonication in buffer A [50 mM Tris-HCl (pH 7.5), 500 mM NaCl, 1 mM EDTA, 5 mM β-mercaptoethanol, 5% (v/v) glycerol and protease inhibitor cocktail (Sigma–Aldrich)]. Tris-HCl buffers were made using 1 M Tris-HCl (pH 7.5) (UltraPure™, Invitrogen). We then added ammonium sulfate to the supernatant to 40% (w/v) saturation while stirring at 4°C for 30 min. We centrifuged the sample, collected the supernatant and subsequently added ammonium sulfate to 50% (w/v) saturation and kept stirring at 4°C for 30 min. We collected the pellet (containing ParB^L5C^ proteins) and dissolved it in buffer B [50 mM Tris-HCl (pH 7.5), 1 mM EDTA and 2 mM β-mercaptoethanol]. Before loading onto a heparin column (GE Healthcare), the sample was diluted in buffer B to achieve a conductivity of 18 mS cm^−1^. We used a linear gradient of buffer B containing 1 M NaCl to elute the protein. After collecting the peak fractions, we repeated the dilution in buffer B to 18 mS cm^−1^ conductivity and loaded it onto HiTrap SP columns (GE Healthcare). For elution, we used a linear gradient of buffer B containing 1 M NaCl. We loaded the collected peak fractions directly onto a Superdex 200–16/600 pg column (GE Healthcare) pre-equilibrated in 50 mM Tris-HCl (pH 7.5), 300 mM NaCl and 1 mM TCEP [Tris-(2-Carboxyethyl)phosphine)] (ThermoFischer Scientific). For fluorescent labeling, we incubated purified ParB^L5C^ variant with Alexa647-maleimide at a 1:2 molar ratio (protein:dye). We incubated the mixture for 15 min on ice, centrifuged it for 10 min and then eluted it from a spin desalting column (Zeba) and flash frozen in liquid nitrogen. We estimated the fluorophore labeling efficiency at 74% for ParB-Alexa647 (resulting in 93% labeled ParB dimers) by an inbuilt function on Nanodrop that measures the protein concentrations from absorbance at 280 nm and dye concentration from the absorbance at peak wavelength (here 650 nm for Alexa647). We used extinction coefficients of ϵ = 270 000 M^−1^ cm^−1^ for Alexa647 and ϵ = 7450 M^−1^ cm^−1^ for ParB protein based on its protein sequence.

**Table 1.** DNA primers used in the presented study for cloning and plasmid construction

Primer name	Sequence 5′ to 3′
STM682	ATTCGGATCCTTATGATTCTCGTTCAGACAAAAGCTC
STM683	ATATACCATGGCTAAAGGCTGTGGAAAAGGGATTAATGCG
CD21	GACCGAGATAGGGTTGAGTG
CD22	CAGGGTCGGAACAGGAGAGC
MT30	CTGCAGGAAGGTTTAAACGCATTTAGG
MT31	TAATACGACTCACTATAGGGAGACGC
MT32	CCTGTAGTCTTCTTAATTAAGACGTCAG
MT33	GTACCAAGTCTTCGAATTCGGATC
MT39	GATCCGAATTCGAAGACTTGGTACGGTCTCATCGTAAAGCTTCTTGATAACGGGGAC
MT40	GACGTCTTAATTAAGAAGACTACAGGGGTCTCAATGGTCCAGTCCCATTTCCCCTATCGC

### Construction and purification of coilable 38 kb DNA*_parS_* construct for fluorescence experiments

To prepare a linear fragment adapted for flow cell experiments, we isolated ∼38 kb plasmid pBS-*parS* via a midiprep using a Qiafilater plasmid midi kit (Qiagen). The large plasmid was constructed from multiple smaller components as described in detail in Tišma *et al.* (17). We digested the pBS-*parS* for 2 h at 37°C using NotI-HF or XhoI restriction enzymes (New England Biolabs) and heat-inactivated for 20 min at 80°C. This resulted in the linear fragment that contains the *parS* site close to the middle of the DNA molecule, more specifically at the 0.4 relative position to the DNA ends. To prepare a linear fragment that would allow the introduction of DNA supercoiling upon the change of intercalating dye concentration, we constructed a fragment carrying handles with multiple biotins at the ends that would torsionally constrain the molecule from rotation around its axis. The handles for the 38 kb construct were made by PCR using primers CD21/CD22 of a 514 bp from the larger template pJT186 [see Table 1 and Tišma *et al.* (17) for details and sequences] in the presence of 1:5 ratio of biotin-16-dUTP (Jena Bioscience, NU-803-BIO16-L) to dTTP (Thermo Fisher Scientific, 10520651). This allows stochastic, multiple insertions of biotinylated nucleotides into the final DNA*_parS_* ends. We digested these biotinylated PCR fragments using NotI-HF or XhoI for 2 h at 37°C, which resulted in the handles of ∼250 bp in length. We mixed the digested handles with the large 38 kb fragment in 10:1 molar ratio (biotin-handles to DNA*_parS_*) and added T4 DNA ligase (New England Biolabs, M0202L) and 1 mM ATP for ligation. The ligation was set overnight at 16°C and subsequently heat-inactivated the next day for 20 min at 65°C. To remove the excess of biotin handles from the large fragment, we used ÄKTA pure, with a homemade gel filtration column containing ∼46 ml of Sephacryl S-1000 SF gel filtration media (Cytiva), run with TE buffer with 150 mM NaCl_2_. The sample was run at a speed of 0.3 ml min^−1^. The collected fractions containing the expected DNA size from the ÄKTA purification were stored at 4°C, until use, in order to avoid freeze–thaw cycles that would introduce nicks into the DNA molecules. The final mixture contains ∼30–40% coilable molecules and the rest non-coilable/nicked, which served as the control comparison throughout this work.

### Single-molecule visualization assay

We performed the experiments with supercoiled DNA and ParB proteins in custom-made flow cells, built by connecting a surface-passivated glass slide and a glass coverslip using double-sided tape (45,46). The surfaces were prepared as described in detail by Chandranoss *et al.* (45) with slight modifications. After extensive cleaning, the surface was silanized using 3-aminopropyl-triethoxysilane (10% v/v) and acetic acid (5% v/v) methanol solution. We passivated the surface with NHS-ester PEG (N-hydroxysuccinimide-ester polyethylene glycol, 5000 Da) and biotinylated NHS-ester PEG (5000 Da) in relation ∼40:1. This step was repeated 4 × 24 h to ensure low adhesion of ParB proteins to the surface. Additionally, we treated the surface with 0.5 mg ml^−1^ UltraPure™ bovine serum albumin (BSA) (Thermo Fisher Scientific) for 30 min in T20 buffer [40 mM Tris-HCl (pH 7.5), 20 mM NaCl]. This further reduced the non-specific adhesion of labeled ParB to the surface.

For immobilization of 38 kb DNA*_parS_*, we introduced 50 μl of ∼3 pM of biotinylated-DNA*_parS_* molecules at a flow rate of 1.5–4 μl min^−1^ in imaging buffer without the oxygen scavenging enzymes [40 mM Tris-HCl (pH 7.5), 65 mM KCl, 2.5 mM MgCl_2_, 2 mM Trolox, 1 mM TCEP, 30 mM glucose, 0.25 mg ml^−1^ BSA, 1 mM CTP, 25–400 nM SYTOX Orange (SxO, Thermo Fisher Scientific)]. Immediately after the flow, we further flowed 100 μl of the wash buffer [40 mM Tris–HCl (pH 7.5), 20 mM NaCl, 65mM KCl, 25–400 nM SxO] at the same flow rate to ensure stretching and tethering of the other end of the DNA to the surface. By adjusting the flow, we obtained a stretch of around 20–50% of the contour length of DNA. The positive and negative supercoiling was induced by changing the SxO concentration during the initial tethering of the DNA*_parS_*and during imaging. Namely, to induce positive supercoiling of the tethered DNA, we tethered the DNA at the initial 25 nM SxO while the final experiments are done in 250 nM SxO in the imaging buffer [40 mM Tris-HCl (pH 7.5), 65 mM KCl, 2.5 mM MgCl_2_, 1 mM CTP, 2 mM Trolox, 1 mM TCEP, 10 nM Catalase, 18.75 nM glucose oxidase, 30 mM glucose, 0.25 μg ml^−1^ BSA, 50–250 nM SxO]. Conversely, to induce negative supercoiling the initial tethering was done in 400 nM SxO while the final imaging is done at 50 nM SxO. The release of prebound SxO dyes after immobilization of the DNA results and now presence of the lower amount of dyes in the DNA results in negative supercoiling of the DNA. The final supercoiling levels within the surface-bound DNA were similar to previously reported levels for bacterial genomes (40) [σ ≈ ±0.05, estimated using a model developed in (47)].

Next, we flowed in the imaging buffer without ParB protein at a very low flow rate (0.2 μl min^−1^) to enable minimal disturbances to the DNA molecules before and after protein addition. Real-time observation of ParB diffusion was carried out by introducing ParB (0.2–25 nM) in the imaging buffer. We used a home-built objective-TIRF microscope with Di01-R405/488/561/635–25 dichroic mirror (BrightLine^®^, IDEX H&S) and NF03-405/488/561/635E-25 quad-notch emission filter (StopLine^®^, IDEX H&S) for fluorescence imaging. We used alternating excitation of 561 nm (0.2 mW) and 647 nm (14 mW) lasers in Highly Inclined and Laminated Optical sheet (HiLo) microscopy mode to image SxO-stained DNA and Alexa647-labeled ParB respectively. HiLo allows imaging of a thin section near the surface with a penetration depth of a few microns, which allows capturing the DNA molecules but reducing the out of focus signal from surrounding fluorophores. All images were acquired with an PrimeBSI sCMOS, Complementary Metal-Oxide Semiconductor, camera at an exposure time of 100 ms (10 Hz frame rate), with a 60× oil immersion, 1.49 NA CFI APO TIRF (Nikon).

### Image processing and analysis

Areas with single DNA molecules were cropped from the raw image sequences using Fiji (48) and analyzed separately with a previously published python software (49,50). Here, the rectangle area around individual molecules were selected and cropped into a new video containing only the individual molecules. This was repeated for all molecules in a single field of view. For the analysis of these regions, the software smoothened the cropped image section using a median filter with a set window size of 10 pixels, and the subtracted the background with the ‘white_tophat’ operation provided in the *scipy* python module (51). We adjusted the contrast of obtained images manually for visualization purposes only (i.e. Figure 4C). The ends of a DNA were manually marked. To get kymographs of our image sequences, we obtained total fluorescence intensity of 11 pixels across the axis of the DNA and stacked them over time axis (i.e. Figure 3B). We chose the exact same DNA axis to obtain kymograph of the ParB fluorescence channel and the frame times were matched to be identical by shifting the second channel by a single frame.

To further analyze the kymographs, we identified the ‘peaks’ for high DNA and protein kymographs by finding the local maximum value within each frame using the *scipy* python module. These maxima were merged into individual tracks if the two maxima are within 7 pixels distance and 5 frames (1 s) away from each other. From these tracks, we calculated the size of a plectoneme or DNA condensate from the fraction of fluorescence intensity in the tracked peak relative to the overall fluorescence intensity of the DNA. The peak pixel was extended by ± 2 pixels to cover the width of the signal. We used an 11-frame moving window to calculate the apparent diffusion constant *D* over time (Figure 4D and H). We calculated the diffusion constants of plectonemes (i.e. Figure 5B) for each analyzed DNA molecule from the MSD calculated over a lag time ranging from 2 to 20 frames, then by fitting to the function $MSD = 2D{\mathrm{\tau }}$, where τ is the lag time. The condensation fraction, as reported in Figure 5A, was calculated from manual identification based on the presence of ParB-Alexa647, a high DNA signal and a low apparent diffusion constant. As DNA plectonemes can be confused for condensed segments of DNA, we screened for both the presence of ParB and significant change in the plectoneme diffusion dynamics (D <10 kb^2^ s^−1^) over an extended time period (200 frames, i.e. 40 s) before classifying an event as ‘supercoiled condensate’ versus just a local diffusing plectoneme. The 1D curves diffusion signal, condensate size signal and ParB intensity signal (Figure 4) were filtered using median filter of the window size of 9 frames. Analyses of the plectoneme size versus DNA position as well as plectoneme position versus ParB cluster position were carried out using custom-written scripts in Igor Pro V6.39 (Wavemetrics, USA).

### Construction of 4.2 kb DNA*_parS_* construct for AFM experiments

To construct the circular DNA for AFM experiments we used a commercially available pGGA plasmid backbone (New England Biolabs). We made a linearized fragment of the pGGA plasmid using a PCR reaction with MT032 and MT033 primers (Table 1), which served as a backbone for the insertion of the *parS*-containing fragment. In parallel to this, we extracted a region containing the *parS* site downstream of *metS* gene in *B. subtilis* genome by a colony PCR using primers MT039 and MT040 (Table 1). We combined the plasmid backbone with the colony PCR insert by mixing them in molar ratio 1:3 in the 2× HiFi mix (New England Biolabs) to obtain the final plasmid of 4175 bp. We incubated the reaction at 50°C for 60 min and cooled it down to 4°C for 30 min. We then transformed 2 μl of this reaction into 50 μl of *E. coli* NEB5alpha cells (New England Biolabs) and verified the presence of insert in grown colonies the following day by sequencing using MT030 and MT031 (Table 1). We grew sequence-positive clones for the plasmid extraction at 37°C overnight in the presence of a selective antibiotic Chloramphenicol (Cm) (30 μg ml^−1^). For obtaining supercoiled plasmids, we diluted the overnight culture 1:100 in 10 ml of fresh LB-Cm (Luria-Bertani) medium and grew at 30°C until the culture reached OD_600_ = 0.6. We then placed the culture on ice for 5 min, and then spun down 4 ml of the culture before proceeding to isolation of the final plasmid using a QIAprep Spin Miniprep kit (Qiagen). The samples were stored at 4°C in order to avoid any freeze–thaw cycles that could introduce nicks into the DNA molecules and lower the yield of supercoiled plasmids. The plasmids that were going to be nicked were extracted directly from the overnight culture. These plasmids were nicked using a modified protocol for Nb. BbvCI nicking enzyme (New England Biolabs) capitalizing on a pre-existing recognition site in the *metS* gene. We added the nicking enzyme (1:50 NEB stock dilution), to the extracted plasmid solution and incubated it at 37°C for 90 min and immediately purified over the PCR extraction membrane (Wizard^®^ SV, Promega) and specifically skipped the recommended 80°C inactivation step, which would result in a fraction of single-stranded DNA in our AFM experiments. The following step was three rounds of plasmid clean-up using the same QIAprep Spin Miniprep kit (Qiagen) to remove all the residual enzymes that could corrupt the AFM images by nonspecifically adhering to the surface.

### AFM experiments and imaging

We obtained images in dry conditions using an AFM from Bruker Multimode 2 (Massachusetts, USA) and Scanassyst-Air-HR tips from Bruker. We incubated samples with different molarity ratios of DNA, CTP and ParB in Eppendorf tubes for 2–5 min in a buffered solution [40 mM Tris (pH 7.5), 70 mM KCl and 7.5 mM MgCl_2_]. Then, we deposited the solution onto a freshly cleaved mica for 30 s. Afterward, we thoroughly washed the surface with 3 ml of Milli-Q water and dried it under a flow of nitrogen until visibly dry. AFM was operated using peak force-tapping mode. We used WSxM software (52) for all image processing and data extraction from our raw data in AFM experiments.

## Results

**Figure 2.**
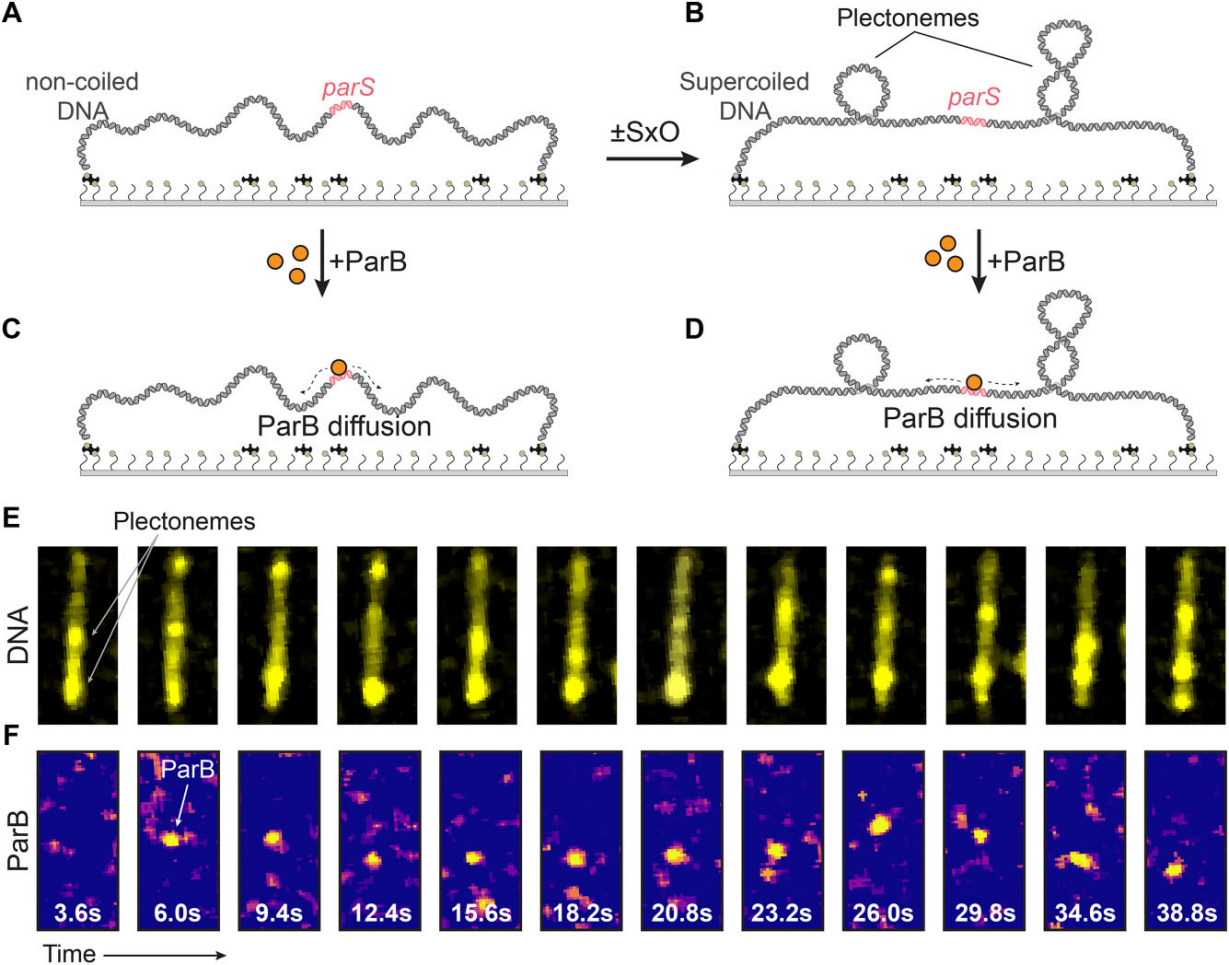
*In vitro* single-molecule fluorescence for studying ParB proteins on supercoiled DNA. (**A**) Schematic representation of the single-molecule DNA stretching assay, with 38 kb DNA*_parS_* tethered to the glass surface. (**B**) The same molecule after the addition or reduction of intercalating dye which induces supercoiling and plectoneme formation. (**C, D**) Same as panels (**A, B**) but with ParB added to the nicked or supercoiled DNA molecules, respectively. (**E**) Images of DNA molecules taken at various times, showing dynamic plectoneme movement on the supercoiled DNA molecule. (**F**) Visualization of single ParB^Alexa647^ dimer on the DNA molecule of panel (**E**), showing binding and 1D diffusion along the DNA*_parS_*at the concentration of 0.1nM.

To observe ParB binding to supercoiled DNA molecules, we employed a single-molecule DNA stretching assay (53) with fluorescently labeled ParB^Alexa647^ proteins (44). We attached a 38 kb DNA molecule that contained a *parS* site close to the middle (DNA*_parS_*) to a glass surface via multiple biotin-streptavidin interactions at both DNA ends (Figure 2A; see the ‘Materials and methods’ section for details). Due to the multiple biotin attachment points at each end, a large fraction of such DNA*_parS_* molecules was torsionally constrained and could not rotate around its central axis to relieve any torsional strain on the molecule. We exploited this feature to directly introduce supercoiling of the desired handedness, either positive or negative supercoiling, to the DNA molecule. We achieved this by changing the concentrations of the intercalator dye SxO that is used for fluorescent visualization of the DNA (46,47,54) (Figure 2B), and does not prevent binding, sliding or DNA condensation by ParB proteins (17,44). When a SxO fluorophore intercalates into the dsDNA helix, it locally pushes two base-pairs apart and thus, due to the induced change of the base-pair distance and angle (55,56), locally underwinds the DNA, which yields an overwinding twist into the remainder of the molecule since the linking number of the molecule is fixed due to the tethering. For sufficiently large SxO concentration, this overwinding yields a positive writhe in the DNA molecule, i.e. positively coiled plectonemes (Figure 2B). The latter were observed as dynamically moving local high-density spots on the linearly stretched DNA molecule (53) (Supplementary Movie S1). The effect of supercoiling on ParB binding and condensation was monitored for both supercoiled and torsionally unconstrained DNA molecules in the same field of view (47) (Supplementary Figure S1).

### ParB proteins efficiently bind and diffuse on supercoiled DNA

**Figure 3.**
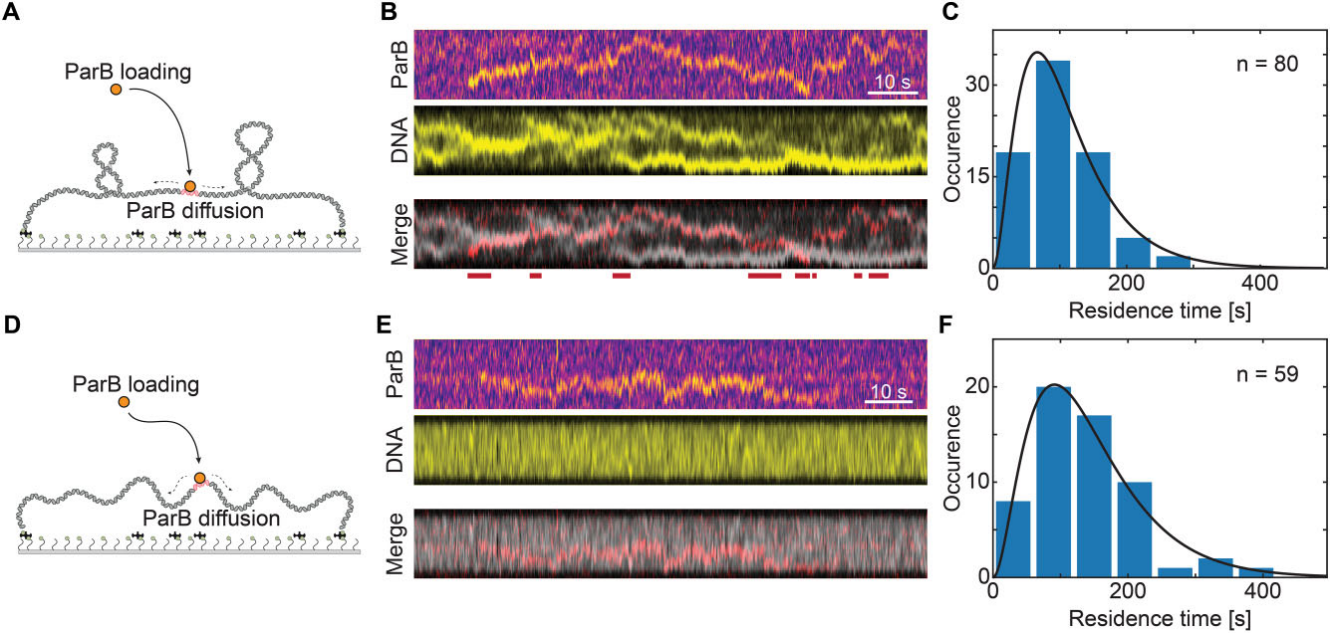
ParB efficiently binds and diffuses along the supercoiled DNA. (**A**) Schematic representation of the supercoiled DNA and addition of ParB proteins. (**B**) Kymographs showing 1D diffusion of a single ParB^Alexa647^ dimers (top) at 0.1nM and DNA plectonemes (middle). Lines below the merged kymograph indicate sections where ParB signal does not overlap the plectonemes. (**C**) Residence times of diffusing ParB dimers after binding to the *parS* site. The data were fitted to a model assuming a delayed dissociation of ParB from the DNA after CTP hydrolysis of both nucleotides [full line, see Tišma *et al.* (44)]. (**D–F**) Same as panels (**A–C**) for non-coiled DNA molecules.

After forming positively supercoiled DNA, we added ParB^Alexa647^ (Figure 2C and D) to observe protein localization and their diffusion behavior along the DNA, similar to our previous report for torsionally unconstrained DNA (44). ParB proteins were observed to load onto the DNA at the *parS* site and to immediately exhibit 1D diffusion away from the binding site, as best observed in a kymograph (Supplementary Figure S2). While ParB diffusion was shown previously on non-coiled DNA molecules (14,24,44), we here observed the efficient loading and diffusion of ParB proteins along the supercoiled DNA molecules. Unexpectedly, we observed that ParB did *not* appear to strongly pin plectonemes (Figure 2E and F). After binding the ParB, the dynamics of the plectonemes continued in an unperturbed way, and no clear co-localization of ParB and the plectonemes was observed (Figure 3A and B, and Supplementary Figure S2B–F). This contrasts many other DNA-binding proteins that were shown to induce a localization of plectonemes at the binding site of the protein (37,57–63), presumably because the binding induced a local change in DNA curvature that lowers the energy of plectoneme formation. We, however, observed continuous 1D diffusion by ParB. ParB dimers that were diffusing on positively supercoiled DNA showed a slightly higher diffusion coefficient D = 0.69 ± 0.35 kb^2^ s^−1^ (median ± SE (standard error); n = 66, Supplementary Figure S3A and B, and Table 2) than on non-coiled DNA molecules (D = 0.43 ± 0.13 kb^2^ s^−1^, median ± SE, n = 58, Figure 3D-F, Supplementary Figure S3C and D).

**Table 2.** Quantification of ParB residence times in the presence of DNA supercoiling

	Mean	Standard error of the mean	Median	Mode	k_CTP_	k_off_	N	Significance	
Non-coiled	141 s	10 s	129 s	91 s	0.016 s^−1^	0.020 ± 0.009 s^−1^	59	MW	KS
Positive supercoiled	109 s	8 s	95 s	70 s	0.017 s^−1^	0.047 ± 0.014 s^−1^	80	0.0028	0.0052
Negative supercoiled	106 s	8 s	100 s	66 s	0.021 s^−1^	0.028 ± 0.016 s^−1^	58	0.0026	0.0072

Significance tests compared to the non-coiled samples: MW and KS. MW and KS are non-parametric statistical tests to assess the difference between two distributions. KS is more sensitive to differences in the distribution shape and spread, while MW is more sensitive to changes in median values. Here, both show high significance *P* < 0.01. KS, Kolmogorov–Smirnov test; MW, Mann–Whitney test.

We observed a non-exponential distribution for the residence time of ParB molecules on DNA (Figure 3C), which contrasts the typical exponential decay for most DNA-binding proteins with the dissociation rate (64,65). This indicates the existence of multiple rate-limiting steps. For ParB, both CTP molecules that are sandwiched between the ParB monomers likely need to be hydrolyzed in order to open and detach the dimer from the DNA_(12,44)_. To describe prolonged diffusion on the DNA molecule, we applied our previously described model (44), which incorporates CTP hydrolysis as the rate-limiting step that extends the ParB diffusion and spreading on the DNA. From the model we obtained the residence time of 70 s (mode of distribution; Figure 3C and Table 2) of ParB molecules diffusing on supercoiled DNA which was significantly lower in comparison to the molecules diffusing on non-coiled DNA (91 s, *P*-value < 0.005; Figure 3F and Table 2).

While all the data presented above were for positively supercoiled DNA, we observed very similar behavior of ParB proteins on negatively supercoiled DNA. We introduced negative supercoiling into the DNA*_parS_* molecules in the same manner described previously, except that we lowered rather than increased the dye concentration after DNA-binding (see the ‘Materials and methods’ section). Subsequent addition of ParB^Alexa647^ molecules showed efficient binding of ParB to negatively supercoiled DNA molecules (Supplementary Figure S4A and B), with a typical residence time of 66 s (mode of distribution; Supplementary Figure S4C and Table 2), and a diffusion coefficient of 0.59 ± 0.25 kb^2^ s^−1^ (median ± SE, Supplementary Figure S4D and E). The results thus show that the presence of DNA supercoiling did not hinder the loading and diffusion of single ParB molecules. However, it did affect the dynamics of diffusing molecules by decreasing the residence time and slightly increasing the diffusion coefficient, albeit with only a marginal level of statistical significance (0.01 < *P* < 0.05), indicating a weak but noteworthy trend.

### Supercoiling facilitates DNA condensation by ParB proteins

In previous *in vivo* and *in vitro* studies, ParB proteins were shown to form ParB–DNA condensates around the *parS* site (14,15,17,22,24,25), and various current models suggest a strong dependence on ParB diffusion and self-self interaction (14–18,20–25). Notably, distant-site binding due to supercoiling-related proximity could severely alter the formation of ParB–DNA condensates, such as was proposed recently by Connolley *et al.* (41).

**Figure 4.**
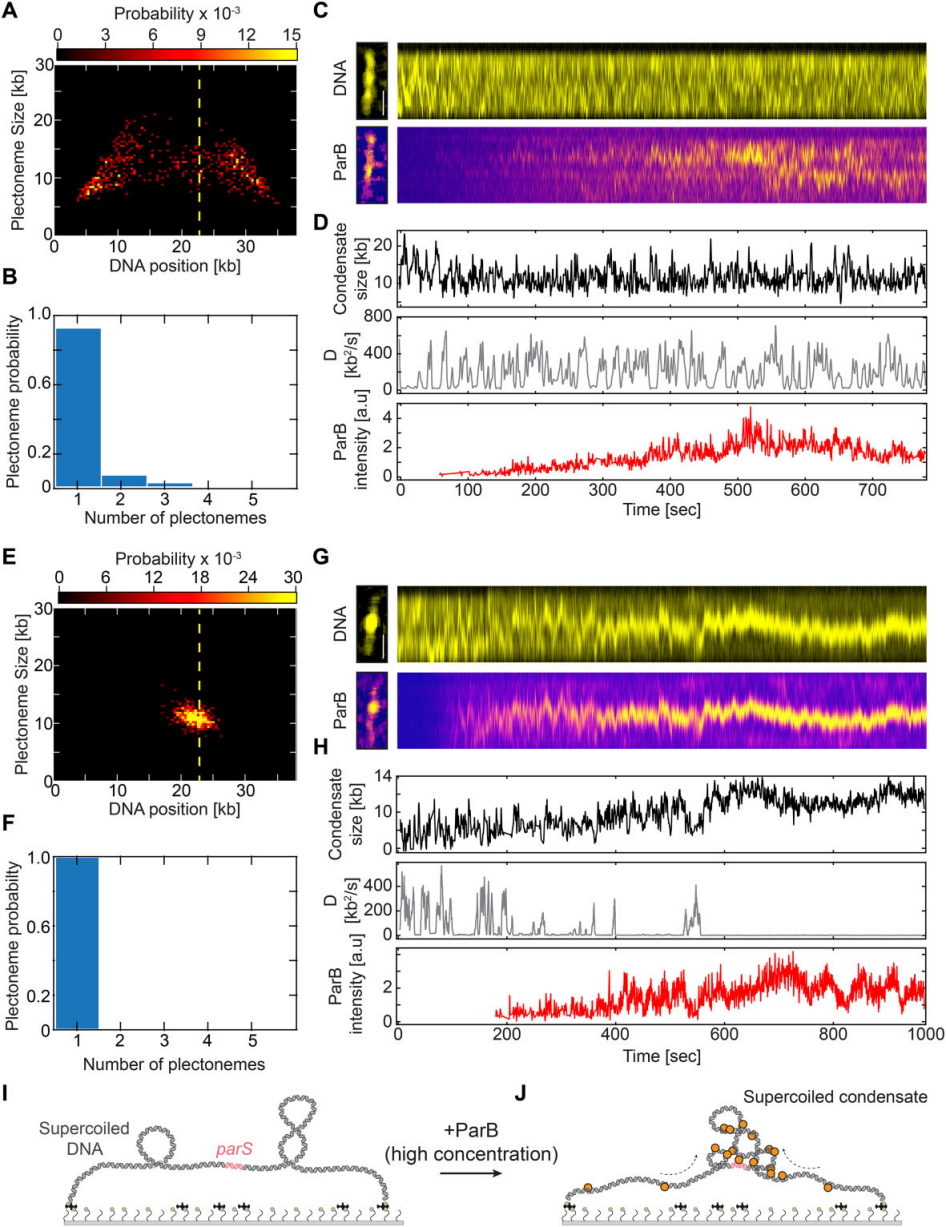
Multiple ParB proteins pin DNA plectonemes into a single static cluster. (**A**) Probability distribution of plectoneme size versus DNA position at 3 nM ParB protein. Yellow dashed line signals the position of the *parS* sequence. (**B**) Observed number of plectonemes on the supercoiled DNA in the presence of ParB. (**C**) Kymographs showing supercoiled DNA (top) and ParB^Alexa647^ proteins (bottom) in the single-molecule assay. (**D**) Quantification of supercoiled plectoneme DNA amount (top), its diffusion coefficient (middle) and ParB^Alexa647^ intensity signal (bottom) over the time of the kymograph show in panel (**C**). (**E–H**) Same as panel (**A–D**) for 25 nM ParB concentration. (**I–J**) Schematic representation of supercoiled condensate in the presence of multiple ParB proteins.

We therefore set out to test the DNA condensation in the presence of DNA supercoiling at higher concentrations of ParB proteins. For comparison, we added either low (3 nM; Figure 4A–D) or high ParB concentrations (25 nM; Figure 4E–H) onto supercoiled DNA*_parS_* molecules. At low ParB concentration, the rapidly moving plectonemes on the DNA largely remained unaffected after ParB addition, both in their position (Figure 4A) and the number of co-existing plectonemes formed on the supercoiled DNA (Figure 4B and Supplementary Figure S5A–E). There also was no significant change in the amount of DNA within each plectoneme, and the total amount of DNA integrated over plectonemes remained at the same value, ∼12 kb for this set amount of supercoiling (Figure 4C and D). Furthermore, small ParB clusters were found to not strictly correlate with the position of the plectonemes on the DNA over time, confirming the absence of significant plectoneme pinning (Supplementary Figure S5F and G). The dynamic behavior of DNA in the presence of low ParB concentration was of comparable dynamics and localization to the control in the absence of the protein (Supplementary Figure S5B and C, and Supplementary Figure S5H and I, respectively).

At high ParB concentrations, however, the behavior was strikingly different. All DNA supercoiling plectonemes were found to converge and pin at one spot, namely the position of the ParB–DNA condensate (Figure 4E and F, and Supplementary Figure S6A–F). No dynamic movement of the condensed spot along the DNA was observed (Figure 4G), which contrasts the data on naked non-supercoiled DNA molecules where the condensate size remained highly variable [as shown previously in (17)]. Quantitative analysis of the amount of DNA within the main plectonemic cluster, showed an increase of the DNA amount within the cluster over time by 40% (from ∼8 kb in the plectonemes before condensation to ∼11.5 kb for the final condensate; Figure 4H). The absence of any plectonemes outside the condensate indicates that the ParB–DNA condensate absorbed all supercoiling writhe into a cluster that we term a ‘supercoiled condensate’. This supercoiled condensate was a static object with a negligibly low diffusion coefficient (∼2 kb^2^ s^−1^) (Figure 4H, middle). Overall, the presence of large numbers of ParB proteins on the DNA drastically changed the dynamics of the supercoiled DNA.

**Figure 5.**
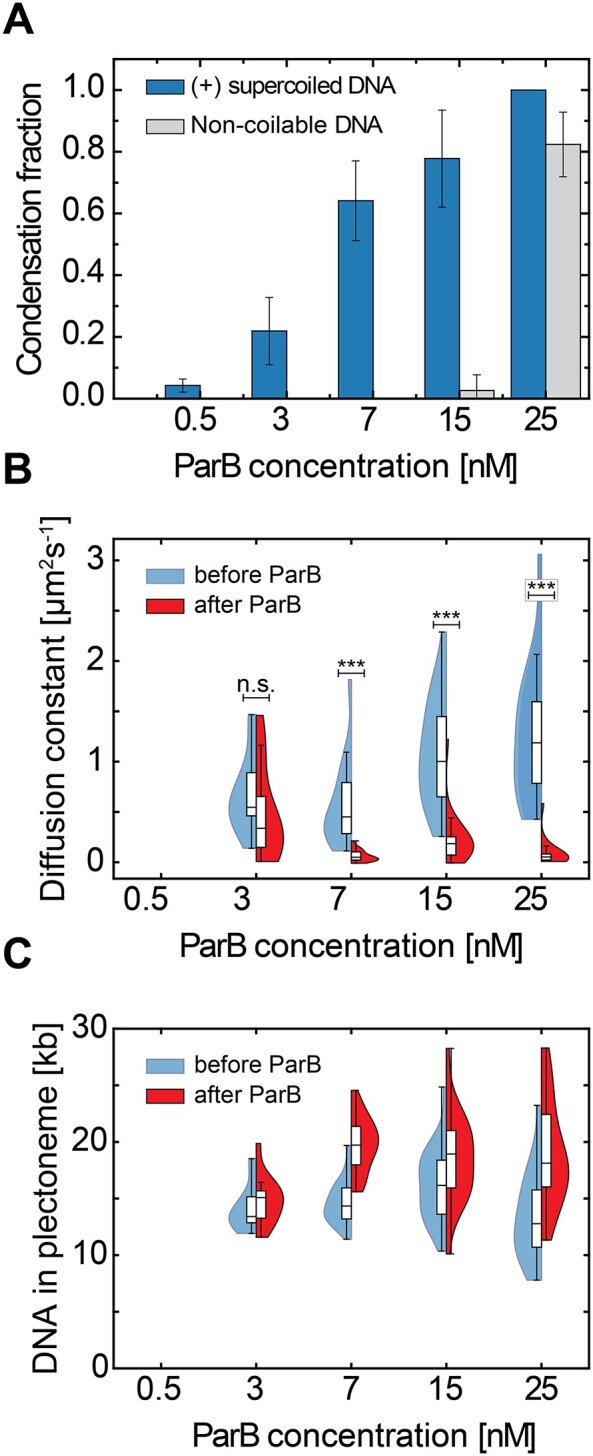
DNA-condensation by ParB proteins is facilitated by DNA supercoiling. (**A**) Fraction of condensed molecules in the presence of an increasing concentration of ParB proteins on non-coiled (gray) and positively supercoiled DNA (blue). Error bars represent the binomial 95% confidence interval. [N: 0.5 nM:0 (no condensed); 3 nM: 12; 7 nM: 34; 15 nM: 21; 25 nM: 6]. (**B**) Diffusion constant of DNA plectonemes or of the supercoiled condensate, measured on the supercoiled molecules shown in panel (**A**) (blue bars) in the presence of ParB proteins. Blue: before ParB enters the flow channel. Red: after > 10 min after ParB was added to the flow channel and is covering the DNA*_parS_* molecules (*P*-values = 0.5 nM: N/A; 3 nM: 0.54; 7 nM: <0.01; 15 nM: 0.19; 25 nM: 0.011). (**C**) Total amount of DNA in plectonemes or in a supercoiled condensate on the 38 kb DNA*_parS_* molecules before (blue) and after (red) the addition of ParB at shown concentration.

To quantify the effects of supercoiling on the ParB–DNA condensation, we screened a range of ParB concentrations (0.5–25 nM) on both negatively and positively supercoiled DNA (Figure 5). We observed that the presence of DNA supercoils did not hinder ParB proteins from spreading all over the DNA molecule at any concentration (Supplementary Figure S7), in line with a recent *in vivo* study (66). Both on non-coiled, negatively and positively supercoiled DNA molecules, the ParB signal spanned over the entire length of the molecule due to diffusion (Supplementary Figure S7).

An important observation was that the presence of DNA supercoiling decreased the minimal ParB concentration that is required for DNA condensation from ∼20 to ∼3 nM. This was observed for both positive (Figure 5A) and negative supercoiling (Supplementary Figure S8A). While torsionally unconstrained DNA molecules did not form condensates at ParB concentrations below ∼25 nM, supercoiled DNA molecules (of either supercoiling sign) showed a sizeable fraction of molecules (∼25%) that exhibited condensation already at 3 nM. The 1D diffusion constant of local DNA spots (i.e. plectonemes or supercoiled condensates at low and high ParB concentrations, respectively) showed a gradual reduction with increased concentrations of ParB (Figure 5B). At the highest concentrations used (25 nM), almost all plectonemes pinned near the middle of the DNA molecules (around the *parS* site) with a diffusion constant close to zero (Figure 5B). This strong reduction in the dynamics of plectonemes was found to be independent of the handedness of the supercoiling, as our data for negative supercoiling (Supplementary Figure S8B) showed the same phenomena as for positive supercoiling (Figure 5B). When the condensed plectonemes pinned onto the DNA, they gradually increased the amount of DNA content within them over time (Figure 4G and H). At the higher ParB concentrations in our experiments, we observed a sizable increase (∼40%) in the average DNA amount within the supercoiled condensate (Figure 5C and Supplementary Figure S8C), albeit this varied between experiments.

### ParB condensate formation collapses linear extended plectonemes

While our data clearly show a pronounced DNA condensation of supercoiled DNA by ParB, the above experiments did not resolve much of the internal structure of the condensate, i.e. whether it is a globular condensed cluster or a linear extended object such as plectoneme. Current models for DNA condensation by ParB proteins propose stochastic bridging interactions of distant segments (14,17) and possible DNA hairpins formed by laddering DNA segments (41).

**Figure 6.**
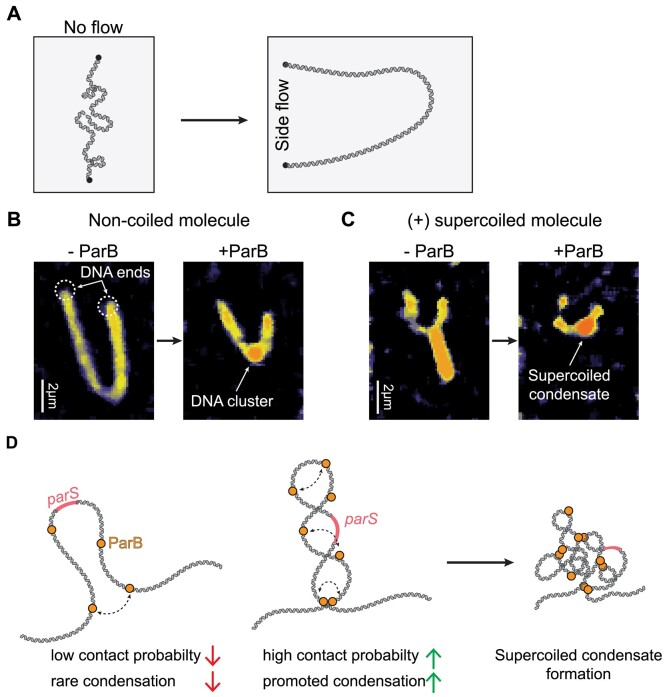
DNA plectoneme is condensed into a compact cluster in the presence of ParB. (**A**) Schematic representation of the side-flow experiment. DNA is tethered parallel to the flow and then a lateral in-plane flow is applied that pushes the DNA in the U-shape only attached with its biotinylated DNA ends to the surface. (**B**) Nicked DNA under side flow in the absence (left) and the presence (right) of 25 nM ParB. (**C**) Same as panel (**B**) but for positively supercoiled DNA molecules. (**D**) Sketches of the molecular conformations upon ParB–DNA condensation in the presence and absence of DNA supercoiling.

To resolve more of the internal structure, we used both our DNA stretching assay and AFM. In our single-molecule visualization assay, we tethered the molecule, induced supercoiling and induced ParB condensation, as in Figure 2. In addition, however, we now exerted an in plane lateral flow that moved the molecule sidewards on the surface, revealing the inner topology of the DNA (Figure 6A). In such experiments with torsionally unconstrained DNA, the lateral flow extended the molecule into a U-shaped arc in the direction of the buffer flow (Figure 6B, left). When doing such experiments after addition of the ParB at high concentration (25 nM), however, the DNA molecules showed a high-intensity condensed spot near the middle of the DNA (Figure 6B, right). This resembles the condensed structure of the partition complex, as reported by multiple works previously (14,15,17,24,25).

In the experiment with supercoiled DNA without ParB, the lateral flow caused instead one long plectonemic structure to align in the flow direction (53) (Figure 6C, left), which presumably results from the merging of multiple dynamic plectonemes into a single long plectoneme. After ParB was loaded on the supercoiled DNA under the same conditions (25 nM), however, the extended structure was found to have collapsed into a non-extended high-intensity spot near the middle of the DNA (Figure 6C, right), which markedly differed from the previous extended plectoneme structure. In fact, the cluster resembled the same shape as the ones on the non-coiled DNA molecules. These images showed that for ParB on supercoiled DNA, the plectonemes were completely condensed into a compact ParB–DNA cluster that had absorbed all supercoiling writhe.

To observe the DNA structure below the optical resolution, we used AFM on 4.2 kb circular DNA*_parS_* that was either supercoiled or nicked. The nicked molecules showed typical open conformations on AFM surface (Supplementary Figure S9A). After the addition of ParB to these non-supercoiled DNA, we observed high compaction in large regions of the DNA molecule or even encompassing the entire DNA molecule (Supplementary Figure S9B) – in line with previous studies (14,17). For supercoiled DNA molecules (without ParB), we observed extended DNA molecules with multiple crossings, typical of plectonemic DNA (Supplementary Figure S9C). Upon addition of ParB to supercoiled DNA molecules, the plasmids showed entirely condensed structures, where any plectonemic regions could not be resolved due to the high compaction and protein coverage in the ParB–DNA clusters (Supplementary Figure S9D). The AFM data confirm the observations of collapsed plectonemic structure in fluorescence assay experiments.

## Discussion

DNA supercoiling is an important regulator of many essential processes such as transcription, replication and DNA compaction and segregation (32,67,68). Vice versa, these processes, e.g. transcription by RNA polymerase, induce supercoiling into the genomic DNA (34,35,37). As a result, bacterial DNA is continuously supercoiled (69,70), both in the bacterial chromosome and in plasmid DNA. Both DNA supercoiling and the ParAB*S* system promote distant intramolecular interactions by, respectively, plectoneme formation (39) and formation of condensed DNA structures by ParB–ParB bridging (14,15,17,23,25,30). In this work, we addressed the question of how these two processes affect one another.

### The impact of DNA supercoiling on ParB

The hallmark behaviors in ParB’s mechanism of action are (i) loading to *parS* site, (ii) clamping and diffusion, and (iii) ParB–ParB bridging which allows DNA condensation. We observed that the presence of DNA supercoiling did not prevent ParB binding, diffusion or condensate formation on the DNA molecules (Figures 2–4), which is in line with recent *in vivo* data on plasmid DNA molecules (66), but it did quantitatively affect the dynamics of diffusion and condensate formation in ParB mechanism. ParB molecules bound efficiently onto negatively supercoiled DNA, non-coiled DNA and positively supercoiled DNA, and all these cases could diffuse along these DNA substrates. No local pinning of plectonemes to locally bound ParB was observed. This could be attributed to the atypical topological binding of ParB to DNA: while most DNA-binding proteins firmly bind to a tight DNA–protein interface, ParB proteins only briefly interact with their *parS* recognition sequence (12,13), whereupon they release from it and topologically encircle the DNA (6,11). Topological entrapment of the DNA likely allows them to freely diffuse along DNA, irrespective of the sequence or twist of the DNA. We observed a somewhat faster diffusion but shorter residence times of ParB in the presence of DNA supercoiling, where interestingly there was no significant difference between different supercoiling handedness (positive and negative). Increased 1D diffusion may be due to an altered affinity of clamped ParB (i.e. after release from *parS*) to supercoiled DNA whereby it can slide faster on the DNA that contains twist, although this difference was marginally significant (Supplementary Figure S4E), indicating that the effect should be interpreted with caution. Alternatively, a direct electrostatic interaction between the C-terminal domain and DNA backbone, which was reported in some species (i.e. *B. subtilis*) (16), may be reduced and thus allow faster diffusion. The decreased residence time is not trivial from the structural point of view, as the CTP binding pocket (N-terminus) of ParB is distant from the lumen that entraps the twisted DNA (C-terminus). While CTP-hydrolysis rates are unaffected, the faster release time of ParB may be due to a lowered affinity of the C-terminus to the supercoiled DNA backbone post-hydrolysis.

While DNA supercoiling does not greatly affect the behavior of single ParB proteins on DNA, we observed that it has a very strong effect on the DNA condensate that is formed by multiple ParB proteins. The concentration required to form a condensate on DNA was strongly (>5-fold) reduced in the presence of DNA supercoiling, which contrasts a previous report using magnetic tweezers (25). Supercoiling thus greatly facilitates the partition complex formation. This is likely to be a result of increased intramolecular interactions between distant DNA segments, which occurs in plectonemic DNA. Here, ParB proteins would load and spread along the DNA (Figure 1) irrespective of the supercoil presence. Upon CTP hydrolysis, ParB proteins open and either dissociate or form a ParB–ParB bridge. In the absence of supercoiling, such bridge formation by ParB proteins would rely on solely on thermal fluctuations of the polymer whereby the probability of distant segments of the DNA molecule meeting is low (Figure 6D). However, with increased intramolecular interactions and proximity of the DNA chains in supercoiled DNA, ParB–ParB bridges can be formed with a higher probability (Figure 6D). Similar observations were made in previous modeling studies that indicated that the introduction of DNA supercoiling promoted the formation of partition complexes (41,42). A recent study by Alaoui *et al.* (66), however, suggested no strong influence of supercoiling on the formation of partition complex in plasmids. This was concluded from ChIP-seq and fluorescence *in vivo* data, whereby it is difficult to deduce the 3D structure of the ParB–DNA complex. Our data corroborate their findings that ParB can still efficiently spread over the supercoiled DNA [similar to ChIP-seq data (66)], independent of the supercoiling handedness. Notably, the same study showed that linearization of the plasmids, close to the *parS* site, increased the plasmid loss fraction by >25-fold. When the plasmid linearization was induced at the larger distances (13 and 47 kb) that ParB proteins, and likely supercoils (71,72), would not reach by diffusion *in vivo*, the plasmid loss was identical to the untreated control. Our data experimentally support that the formation of the 3D partition complex is strongly promoted by DNA supercoiling, and we hypothesize that this may be particularly important right after the origin replication when the available ParB concentration outside of the partition complex is very low (23).

### The impact of ParB on supercoiled DNA

While supercoiling strongly affected the condensation of ParB proteins on DNA, ParB also drastically changed the dynamics of supercoiled DNA. As DNA condensation by ParB proteins proceeded, the motion of the rapidly moving plectonemes slowed down, until finally a single static spot on the DNA emerged (Figures 4 and 5). We termed this structure a ‘supercoiled condensate’ as it is a ParB–DNA condensate that absorbed all writhe, i.e. all plectonemic supercoils. The change in the dynamics of DNA plectonemes was similarly present in both positive and negatively supercoiled DNA, likely because this effect is induced by the interwinding plectonemic structure which facilitates ParB–ParB bridging. As a result of the ParB interactions, the supercoiled condensate lost the characteristic linearly extended plectonemic structure (Figure 6). Notably, the data show that the ParB partition complex can act as a topological barrier to supercoiling, as it pins all the nearby supercoils into the condensed structure.

Interestingly, previous work showed that brief rifampicin treatment of bacterial cells showed a complete loss of any higher-order organization within the origin region (73–75), which is surprising as ParB proteins should have been unaffected in their ability to locally condense the DNA in the partition complex. This observation is consistent with the hypothesis that supercoiling is a crucial facilitator for the maintenance of the ParB–DNA partition complex, especially considering the small number of ParB proteins (∼250–700) in a bacterial cell (23,30). In fact, supercoiling appears to underlie most of the large-scale chromosome compaction in bacterial cells (76), and – as we show here – partition complex formation as well.

The ParABS system also facilitates the segregation and propagation of many plasmids in bacterial cells (77,78). Plasmids are often supercoiled due to continuous high expression of genes that enable their survival, and consecutive replication cycles (79), and for their small sizes [∼1–100 kb (80)] supercoiling often has a significant effect on the entire molecule rather than on a local fraction. The effect of facilitated partition complex formation in the presence of DNA supercoiling therefore also has significant implications for plasmid biology as their segregation to daughter cells could be impacted by their supercoiling density which is determined by the level of transcription and phase of the cell cycle.

Overall, this study provides interesting mechanistic insights into how two essential processes within the bacterial cells, DNA supercoiling and the DNA segregation machinery, interact.

## Supplementary Material

gkae936_Supplemental_Files

## Data Availability

All raw data from experiments are available upon request to corresponding authors.

## References

[1] Jalal A.S.B. , Le T.B.K. Bacterial chromosome segregation by the ParABS system. Open Biology. 2020; 10:200097. 32543349 10.1098/rsob.200097 PMC7333895

[2] Livny J. , Yamaichi Y., Waldor M.K. Distribution of centromere-like parS sites in bacteria: insights from comparative genomics. J. Bacteriol. 2007; 189:8693–8703. 17905987 10.1128/JB.01239-07 PMC2168934

[3] Davis M.A. , Martin K.A., Austin S.J. Biochemical activities of the parA partition protein of the P1 plasmid. Mol. Microbiol. 1992; 6:1141–1147. 1534133 10.1111/j.1365-2958.1992.tb01552.x

[4] Watanabe E. , Wachi M., Yamasaki M., Nagai K. ATPase activity of SopA, a protein essential for active partitioning of F plasmid. Mol. Gen. Genet. 1992; 234:346–352. 1406581 10.1007/BF00538693

[5] Osorio-Valeriano M. , Altegoer F., Steinchen W., Urban S., Liu Y., Bange G., Thanbichler M. ParB-type DNA segregation proteins are CTP-dependent molecular switches. Cell. 2019; 179:1512–1524. 31835030 10.1016/j.cell.2019.11.015

[6] Soh Y.M. , Davidson I.F., Zamuner S., Basquin J., Bock F.P., Taschner M., Veening J.W., de Los Rios P., Peters J.M., Gruber S. Self-organization of parS centromeres by the ParB CTP hydrolase. Science. 2019; 366:1129–1133. 31649139 10.1126/science.aay3965 PMC6927813

[7] Lin D.C.H. , Grossman A.D. Identification and characterization of a bacterial chromosome partitioning site. Cell. 1998; 92:675–685. 9506522 10.1016/s0092-8674(00)81135-6

[8] Corrales-Guerrero L. , He B., Refes Y., Panis G., Bange G., Viollier P.H., Steinchen W., Thanbichler M. Molecular architecture of the DNA-binding sites of the P-loop ATPases MipZ and ParA from Caulobacter crescentus. Nucleic Acids Res. 2020; 48:4769–4779. 32232335 10.1093/nar/gkaa192 PMC7229837

[9] Zhang H. , Schumacher M.A. Structures of partition protein ParA with nonspecific DNA and ParB effector reveal molecular insights into principles governing Walker-box DNA segregation. Genes Dev. 2017; 31:481–492. 28373206 10.1101/gad.296319.117 PMC5393062

[10] Jalal A.S. , Tran N.T., Le T.B. ParB spreading on DNA requires cytidine triphosphate in vitro. eLife. 2020; 9:e53515. 32077854 10.7554/eLife.53515 PMC7053999

[11] Osorio-Valeriano M. , Altegoer F., Das C.K., Steinchen W., Panis G., Connolley L., Giacomelli G., Feddersen H., Corrales-Guerrero L., Giammarinaro P.I. et al. The CTPase activity of ParB determines the size and dynamics of prokaryotic DNA partition complexes. Mol. Cell. 2021; 81:3992–4007. 34562373 10.1016/j.molcel.2021.09.004

[12] Antar H. , Soh Y.-M., Zamuer S., Bock F.P., Anchimiuk A., De Los Rios P., Gruber S. Relief of ParB autoinhibition by parS DNA catalysis and ParB recycling by CTP hydrolysis promote bacterial centromere assembly. Sci. Adv. 2021; 7:eabj2854. 34613769 10.1126/sciadv.abj2854 PMC8494293

[13] Jalal A.S.B. , Tran N.T., Stevenson C.E.M., Chimthanawala A., Badrinarayanan A., Lawson D.M., Le T.B.K. A CTP-dependent gating mechanism enables ParB spreading on DNA. eLife. 2021; 10:e69676. 34397383 10.7554/eLife.69676 PMC8367383

[14] Balaguer F.d.A. , Aicart-Ramos C., Fisher G.L.M., de Bragança S., Martin-Cuevas E.M., Pastrana C.L., Dillingham M.S., Moreno-Herrero F. CTP promotes efficient ParB-dependent DNA condensation by facilitating one-dimensional diffusion from parS. eLife. 2021; 10:e67554. 34250901 10.7554/eLife.67554 PMC8299390

[15] Graham T.G.W. , Wang X., Song D., Etson C.M., van Oijen A.M., Rudner D.Z., Loparo J.J. ParB spreading requires DNA bridging. Genes Dev. 2014; 28:1228–1238. 24829297 10.1101/gad.242206.114 PMC4052768

[16] Taylor J.A. , Pastrana C.L., Butterer A., Pernstich C., Gwynn E.J., Sobott F., Moreno-Herrero F., Dillingham M.S. Specific and non-specific interactions of ParB with DNA: implications for chromosome segregation. Nucleic Acids Res. 2015; 43:719–731. 25572315 10.1093/nar/gku1295 PMC4333373

[17] Tišma M. , Janissen R., Antar H., Martin-Gonzalez A., Barth R., Beekman T., van der Torre J., Michieletto D., Gruber S., Dekker C. Dynamic ParB–DNA interactions initiate and maintain a partition condensate for bacterial chromosome segregation. Nucleic Acids Res. 2023; 51:11856–11875. 37850647 10.1093/nar/gkad868 PMC10681803

[18] Broedersz C.P. , Wang X., Meir Y., Loparo J.J., Rudner D.Z., Wingreen N.S. Condensation and localization of the partitioning protein ParB on the bacterial chromosome. Proc. Natl Acad. Sci. U.S.A. 2014; 111:8809–8814. 24927534 10.1073/pnas.1402529111 PMC4066521

[19] Funnell B.E. ParB partition proteins: complex formation and spreading at bacterial and plasmid centromeres. Front. Mol. Biosci. 2016; 3:44. 27622187 10.3389/fmolb.2016.00044 PMC5002424

[20] Sanchez A. , Cattoni D.I., Walter J.C., Rech J., Parmeggiani A., Nollmann M., Bouet J.Y. Stochastic self-assembly of ParB proteins builds the bacterial DNA segregation apparatus. Cell Syst. 2015; 1:163–173. 27135801 10.1016/j.cels.2015.07.013

[21] Debaugny R.E. , Sanchez A., Rech J., Labourdette D., Dorignac J., Geniet F., Palmeri J., Parmeggiani A., Boudsocq F., Anton Leberre V. et al. A conserved mechanism drives partition complex assembly on bacterial chromosomes and plasmids. Mol. Syst. Biol. 2018; 14:e8516. 30446599 10.15252/msb.20188516 PMC6238139

[22] Babl L. , Giacomelli G., Ramm B., Gelmroth A.K., Bramkamp M., Schwille P. CTP-controlled liquid–liquid phase separation of ParB. J. Mol. Biol. 2022; 434:167401. 34902429 10.1016/j.jmb.2021.167401

[23] Guilhas B. , Walter J.-C., Rech J., David G., Walliser N., Palmeri J., Mathieu-Demaziere C., Parmeggiani A., Bouet J.-Y., Le Gall A. et al. ATP-driven separation of liquid phase condensates in bacteria. Mol. Cell. 2019; 79:293–303. 10.1016/j.molcel.2020.06.034 32679076

[24] Guo L. , Zhao Y., Zhang Q., Feng Y., Bi L., Zhang X., Wang T., Liu C., Ma H., Sun B. Stochastically multimerized ParB orchestrates DNA assembly as unveiled by single-molecule analysis. Nucleic Acids Res. 2022; 50:9294–9305. 35904809 10.1093/nar/gkac651 PMC9458438

[25] Taylor J.A. , Seol Y., Budhathoki J., Neuman K.C., Mizuuchi K. CTP and parS coordinate ParB partition complex dynamics and ParA-ATPase activation for ParABS-mediated DNA partitioning. eLife. 2021; 10:e65651. 34286695 10.7554/eLife.65651 PMC8357417

[26] Bock F.P. , Liu H.W., Anchimiuk A., Diebold-Durand M.-L., Gruber S. A joint-ParB interface promotes Smc DNA recruitment. Cell Rep. 2022; 40:111273. 36044845 10.1016/j.celrep.2022.111273 PMC9449133

[27] Gruber S. , Errington J. Recruitment of condensin to replication origin regions by ParB/SpoOJ promotes chromosome segregation in *B. subtilis*. Cell. 2009; 137:685–696. 19450516 10.1016/j.cell.2009.02.035

[28] Sullivan N.L. , Marquis K.A., Rudner D.Z. Recruitment of SMC by ParB-parS organizes the origin region and promotes efficient chromosome segregation. Cell. 2009; 137:697–707. 19450517 10.1016/j.cell.2009.04.044 PMC2892783

[29] Hu L. , Vecchiarelli A.G., Mizuuchi K., Neuman K.C., Liu J. Directed and persistent movement arises from mechanochemistry of the ParA/ParB system. Proc. Natl Acad. Sci. U.S.A. 2015; 112:E7055–E7064. 26647183 10.1073/pnas.1505147112 PMC4697391

[30] Lim H.C. , Surovtsev I.V., Beltran B.G., Huang F., Bewersdorf J., Jacobs-Wagner C. Evidence for a DNA-relay mechanism in ParABS-mediated chromosome segregation. eLife. 2014; 3:e02758. 24859756 10.7554/eLife.02758 PMC4067530

[31] Vecchiarelli A.G. , Neuman K.C., Mizuuchi K. A propagating ATPase gradient drives transport of surface-confined cellular cargo. Proc. Natl Acad. Sci. U.S.A. 2014; 111:4880–4885. 24567408 10.1073/pnas.1401025111 PMC3977271

[32] Dorman C.J. , Dorman M.J. DNA supercoiling is a fundamental regulatory principle in the control of bacterial gene expression. Biophys. Rev. 2016; 8:209–220. 28510224 10.1007/s12551-016-0205-y PMC5425793

[33] Lal A. , Dhar A., Trostel A., Kouzine F., Seshasayee A.S., Adhya S. Genome scale patterns of supercoiling in a bacterial chromosome. Nat. Commun. 2016; 7:11055. 27025941 10.1038/ncomms11055 PMC4820846

[34] Liu L.F. , Wang J.C. Supercoiling of the DNA template during transcription. Proc. Natl Acad. Sci. U.S.A. 1987; 84:7024–7027. 2823250 10.1073/pnas.84.20.7024 PMC299221

[35] Ma J. , Wang M.D. DNA supercoiling during transcription. Biophys. Rev. 2016; 8:75–87. 10.1007/s12551-016-0215-9 PMC5338639 28275417

[36] Tsao Y.P. , Wu H.Y., Liu L.F. Transcription-driven supercoiling of DNA: direct biochemical evidence from in vitro studies. Cell. 1989; 56:111–118. 2535966 10.1016/0092-8674(89)90989-6

[37] Janissen R. , Barth R., Polinder M., van der Torre J., Dekker C. Single-molecule visualization of twin-supercoiled domains generated during transcription. Nucleic Acids Res. 2024; 52:1677–1687. 38084930 10.1093/nar/gkad1181 PMC10899792

[38] Boles T.C. , White J.H., Cozzarelli N.R. Structure of plectonemically supercoiled DNA. J. Mol. Biol. 1990; 213:931–951. 2359128 10.1016/S0022-2836(05)80272-4

[39] Zuccheri G. , Dame R.T., Aquila M., Muzzalupo I., Samorì B. Conformational fluctuations of supercoiled DNA molecules observed in real time with a scanning force microscope. Appl. Phys. A. 1998; 66:S585–S589.

[40] Junier I. , Ghobadpour E., Espeli O., Everaers R. DNA supercoiling in bacteria: state of play and challenges from a viewpoint of physics based modeling. Front. Microbiol. 2023; 14:1192831. 37965550 10.3389/fmicb.2023.1192831 PMC10642903

[41] Connolley L. , Schnabel L., Thanbichler M., Murray S.M. Partition complex structure can arise from sliding and bridging of ParB dimers. Nat. Commun. 2023; 14:4567. 37516778 10.1038/s41467-023-40320-y PMC10387095

[42] Walter J.C. , Lepage T., Dorignac J., Geniet F., Parmeggiani A., Palmeri J., Bouet J.Y., Junier I. Supercoiled DNA and non-equilibrium formation of protein complexes: a quantitative model of the nucleoprotein ParBS partition complex. PLoS Comput. Biol. 2021; 17:e1008869. 33861734 10.1371/journal.pcbi.1008869 PMC8092679

[43] Tišma M. , Kaljević J., Gruber S., Le T.B.K., Dekker C. Connecting the dots: key insights on ParB for chromosome segregation from single-molecule studies. FEMS Microbiol. Rev. 2024; 48:fuad067. 38142222 10.1093/femsre/fuad067 PMC10786196

[44] Tišma M. , Panoukidou M., Antar H., Soh Y.-M., Barth R., Pradhan B., Barth A., van der Torre J., Michieletto D., Gruber S. et al. ParB proteins can bypass DNA-bound roadblocks via dimer-dimer recruitment. Sci. Adv. 2022; 8:eabn3299. 35767606 10.1126/sciadv.abn3299 PMC9242446

[45] Chandradoss S.D. , Haagsma A.C., Lee Y.K., Hwang J.H., Nam J.M., Joo C. Surface passivation for single-molecule protein studies. J. Vis. Exp. 2014; 86:50549. 10.3791/50549 PMC4179479 24797261

[46] Ganji M. , Kim S.H., van der Torre J., Abbondanzieri E., Dekker C. Intercalation-based single-molecule fluorescence assay to study DNA supercoil dynamics. Nano Lett. 2016; 16:4699–4707. 27356180 10.1021/acs.nanolett.6b02213

[47] Kolbeck P.J. , Tišma M., Analikwu B.T., Vanderlinden W., Dekker C., Lipfert J. Supercoiling-dependent DNA binding: quantitative modeling and applications to bulk and single-molecule experiments. Nucleic Acids Res. 2024; 52:59–72. 38000393 10.1093/nar/gkad1055 PMC10783501

[48] Schindelin J. , Arganda-Carreras I., Frise E., Kaynig V., Longair M., Pietzsch T., Preibisch S., Rueden C., Saalfeld S., Schmid B. et al. Fiji: an open-source platform for biological-image analysis. Nat. Methods. 2012; 9:676–682. 22743772 10.1038/nmeth.2019 PMC3855844

[49] Pradhan B. , Barth R., Kim E., Davidson I.F., Bauer B., van Laar T., Yang W., Ryu J.-K., van der Torre J., Peters J.-M. et al. SMC complexes can traverse physical roadblocks bigger than their ring size. Cell Rep. 2022; 41:111491. 36261017 10.1016/j.celrep.2022.111491

[50] Pradhan B. , Kanno T., Umeda Igarashi M., Loke M.S., Baaske M.D., Wong J.S.K., Jeppsson K., Björkegren C., Kim E. The Smc5/6 complex is a DNA loop-extruding motor. Nature. 2023; 616:843–848. 37076626 10.1038/s41586-023-05963-3 PMC10132971

[51] Virtanen P. , Gommers R., Oliphant T.E., Haberland M., Reddy T., Cournapeau D., Burovski E., Peterson P., Weckesser W., Bright J. et al. SciPy 1.0: fundamental algorithms for scientific computing in Python. Nat. Methods. 2020; 17:261–272. 32015543 10.1038/s41592-019-0686-2 PMC7056644

[52] Horcas I. , Fernández R., Gómez-Rodríguez J.M., Colchero J., Gómez-Herrero J., Baro A.M. WSXM: a software for scanning probe microscopy and a tool for nanotechnology. Rev. Sci. Instrum. 2007; 78:13705. 10.1063/1.2432410 17503926

[53] Ganji M. , Kim S.H., van der Torre J., Abbondanzieri E., Dekker C. Intercalation-based single-molecule fluorescence assay to study DNA supercoil dynamics. Nano Lett. 2016; 16:4699–4707. 27356180 10.1021/acs.nanolett.6b02213

[54] Kim S.H. , Ganji M., Kim E., van der Torre J., Abbondanzieri E., Dekker C. DNA sequence encodes the position of DNA supercoils. eLife. 2018; 7:e36557. 30523779 10.7554/eLife.36557 PMC6301789

[55] Wang J.C. The degree of unwinding of the DNA helix by ethidium. I. Titration of twisted PM2 DNA molecules in alkaline cesium chloride density gradients. J. Mol. Biol. 1974; 89:783–801. 4449133 10.1016/0022-2836(74)90053-9

[56] Yan X. , Habbersett R.C., Cordek J.M., Nolan J.P., Yoshida T.M., Jett J.H., Marrone B.L. Development of a mechanism-based, DNA staining protocol using SYTOX Orange nucleic acid stain and DNA fragment sizing flow cytometry. Anal. Biochem. 2000; 286:138–148. 11038284 10.1006/abio.2000.4789

[57] Figueroa-Bossi N. , Fernández-Fernández R., Kerboriou P., Bouloc P., Casadesús J., Sánchez-Romero M.A., Bossi L. Transcription-driven DNA supercoiling counteracts H-NS-mediated gene silencing in bacterial chromatin. Nat. Commun. 2024; 15:2787. 38555352 10.1038/s41467-024-47114-w PMC10981669

[58] Jeppsson K. , Pradhan B., Sutani T., Sakata T., Umeda Igarashi M., Berta D.G., Kanno T., Nakato R., Shirahige K., Kim E. et al. Loop-extruding Smc5/6 organizes transcription-induced positive DNA supercoils. Mol. Cell. 2024; 84:867–882.e865. 38295804 10.1016/j.molcel.2024.01.005

[59] Kim E. , Gonzalez A.M., Pradhan B., van der Torre J., Dekker C. Condensin-driven loop extrusion on supercoiled DNA. Nat. Struct. Mol. Biol. 2022; 29:719–727. 35835864 10.1038/s41594-022-00802-x

[60] Mondal A. , Sangeeta, Bhattacherjee A. Torsional behaviour of supercoiled DNA regulates recognition of architectural protein fis on minicircle DNA. Nucleic Acids Res. 2022; 50:6671–6686.

[61] Shahu S. , Vtyurina N., Das M., Meyer A.S., Ganji M., Abbondanzieri E.A. Bridging DNA contacts allow dps from *E. coli* to condense DNA. 2024; bioRxiv doi:25 January 2024, preprint: not peer reviewed 10.1101/2024.01.22.576774. PMC11077075 38572752

[62] Tanaka H. , Yasuzawa K., Kohno K., Goshima N., Kano Y., Saiki T., Imamoto F. Role of HU proteins in forming and constraining supercoils of chromosomal DNA in Escherichia coli. Mol. Gen. Genet. 1995; 248:518–526. 7476850 10.1007/BF02423446

[63] Watson G.D. , Chan E.W., Leake M.C., Noy A. Structural interplay between DNA-shape protein recognition and supercoiling: the case of IHF. Comput. Struct. Biotechnol. J. 2022; 20:5264–5274. 36212531 10.1016/j.csbj.2022.09.020 PMC9519438

[64] Chen T.Y. , Cheng Y.S., Huang P.S., Chen P. Facilitated unbinding via multivalency-enabled ternary complexes: new paradigm for protein–DNA interactions. Acc. Chem. Res. 2018; 51:860–868. 29368512 10.1021/acs.accounts.7b00541 PMC5904000

[65] Kamar R.I. , Banigan E.J., Erbas A., Giuntoli R.D., Olvera de la Cruz M., Johnson R.C., Marko J.F. Facilitated dissociation of transcription factors from single DNA binding sites. Proc. Natl Acad. Sci. U.S.A. 2017; 114:E3251–E3257. 28364020 10.1073/pnas.1701884114 PMC5402408

[66] Alaoui H.S. , Quèbre V., Delimi L., Rech J., Debaugny-Diaz R., Labourdette D., Campos M., Cornet F., Walter J.C., Bouet J.Y. *In vivo* assembly of bacterial partition condensates on circular supercoiled and linear DNA. Mol. Microbiol. 2024; 10.1111/mmi.15297. 39109686

[67] Dorman C.J. DNA supercoiling and transcription in bacteria: a two-way street. BMC Mol. Cell Biol. 2019; 20:26. 31319794 10.1186/s12860-019-0211-6 PMC6639932

[68] El Houdaigui B. , Forquet R., Hindré T., Schneider D., Nasser W., Reverchon S., Meyer S. Bacterial genome architecture shapes global transcriptional regulation by DNA supercoiling. Nucleic Acids Res. 2019; 47:5648–5657. 31216038 10.1093/nar/gkz300 PMC6582348

[69] Travers A. , Muskhelishvili G. Bacterial chromatin. Curr. Opin. Genet. Dev. 2005; 15:507–514. 16099644 10.1016/j.gde.2005.08.006

[70] Visser B.J. , Sharma S., Chen P.J., McMullin A.B., Bates M.L., Bates D. Psoralen mapping reveals a bacterial genome supercoiling landscape dominated by transcription. Nucleic Acids Res. 2022; 50:4436–4449. 35420137 10.1093/nar/gkac244 PMC9071471

[71] Deng S. , Stein R.A., Higgins N.P. Transcription-induced barriers to supercoil diffusion in the *Salmonella typhimurium* chromosome. Proc. Natl Acad. Sci. U.S.A. 2004; 101:3398–3403. 14993611 10.1073/pnas.0307550101 PMC373473

[72] Postow L. , Hardy C.D., Arsuaga J., Cozzarelli N.R. Topological domain structure of the *Escherichia coli* chromosome. Genes Dev. 2004; 18:1766–1779. 15256503 10.1101/gad.1207504 PMC478196

[73] Wang X. , Brandão H.B., Le T.B.K., Laub M.T., Rudner D.Z. *Bacillus subtilis* SMC complexes juxtapose chromosome arms as they travel from origin to terminus. Science. 2017; 355:524–527. 28154080 10.1126/science.aai8982 PMC5484144

[74] Tišma M. , Bock F.P., Kerssemakers J., Antar H., Japaridze A., Gruber S., Dekker C. Direct observation of a crescent-shape chromosome in expanded *Bacillus subtilis* cells. Nat. Commun. 2024; 15:2737. 38548820 10.1038/s41467-024-47094-x PMC10979009

[75] Marbouty M. , Le Gall A., Cattoni D.I., Cournac A., Koh A., Fiche J.B., Mozziconacci J., Murray H., Koszul R., Nollmann M. Condensin- and replication-mediated bacterial chromosome folding and origin condensation revealed by hi-C and super-resolution imaging. Mol. Cell. 2015; 59:588–602. 26295962 10.1016/j.molcel.2015.07.020

[76] Bignaud A. , Cockram C., Borde C., Groseille J., Allemand E., Thierry A., Marbouty M., Mozziconacci J., Espéli O., Koszul R. Transcription-induced domains form the elementary constraining building blocks of bacterial chromosomes. Nat. Struct. Mol. Biol. 2024; 31:489–497. 38177686 10.1038/s41594-023-01178-2 PMC10948358

[77] Gerdes K. , Møller-Jensen J., Bugge Jensen R. Plasmid and chromosome partitioning: surprises from phylogeny. Mol. Microbiol. 2000; 37:455–466. 10931339 10.1046/j.1365-2958.2000.01975.x

[78] Köhler R. , Kaganovitch E., Murray S.M. High-throughput imaging and quantitative analysis uncovers the nature of plasmid positioning by ParABS. eLife. 2022; 11:e78743. 36374535 10.7554/eLife.78743 PMC9662831

[79] Zechiedrich E.L. , Khodursky A.B., Bachellier S., Schneider R., Chen D., Lilley D.M.J., Cozzarelli N.R. Roles of topoisomerases in maintaining steady-state DNA supercoiling in Escherichia coli. J. Biol. Chem. 2000; 275:8103–8113. 10713132 10.1074/jbc.275.11.8103

[80] Shintani M. , Sanchez Z.K., Kimbara K. Genomics of microbial plasmids: classification and identification based on replication and transfer systems and host taxonomy. Front. Microbiol. 2015; 6:242. 25873913 10.3389/fmicb.2015.00242 PMC4379921

