# Supplementary material for: DNA supercoiling enhances DNA condensation by ParB proteins
Source: Nucleic Acids Res. 2024 Oct 23;52(21):13255–68. doi: 10.1093/nar/gkae936 (PMC11602141; doi:10.1093/nar/gkae936)
Supplement: gkae936_Supplemental_Files [file gkae936_supplemental_files.zip › Martin-Gonzalez et al_Supp_Figures.pdf]

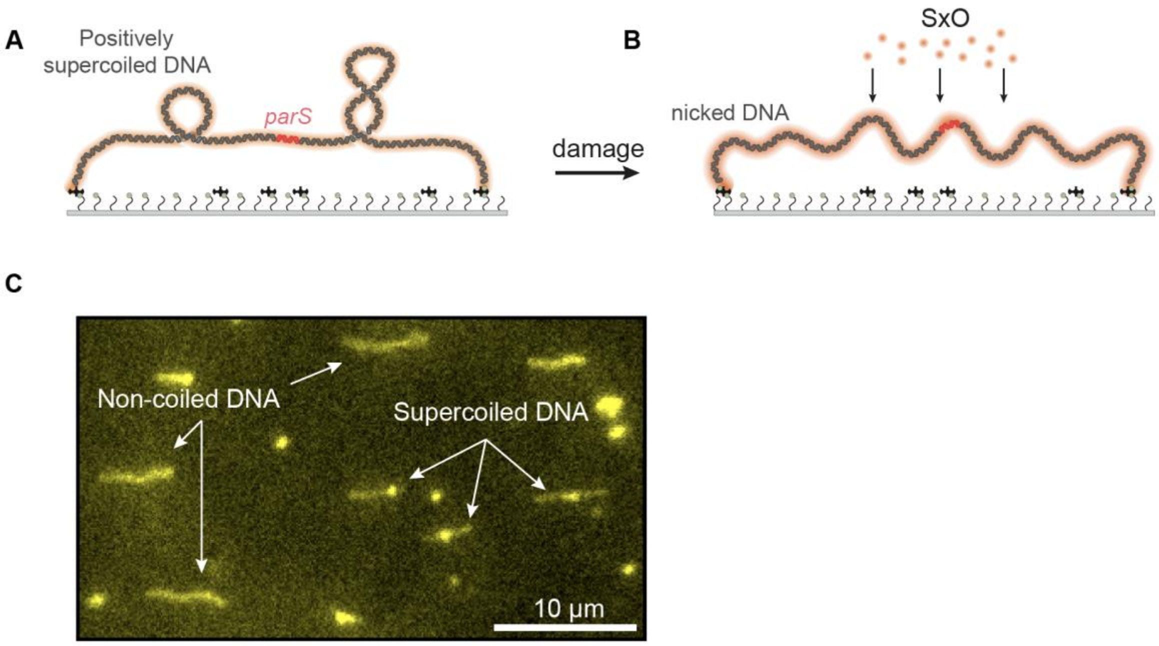

Figure S1. *In vitro* single-molecule fluorescence detection of non-coiled DNA as well as plectonemes on supercoiled DNA. A) Schematic representation of the single-molecule DNA stretching assay, with 38 kbp DNA$_{parS}$ tethered to the glass surface in the presence of SYTOX Orange intercalating dye. **B)** The same molecule if rendered non-coilable, which may e.g. occur due to nicking. Non-coiled DNA molecules appear brighter and more flexible. **C)** Typical section of the experimental field of view with both non-coilable and supercoiled molecules under the same conditions.

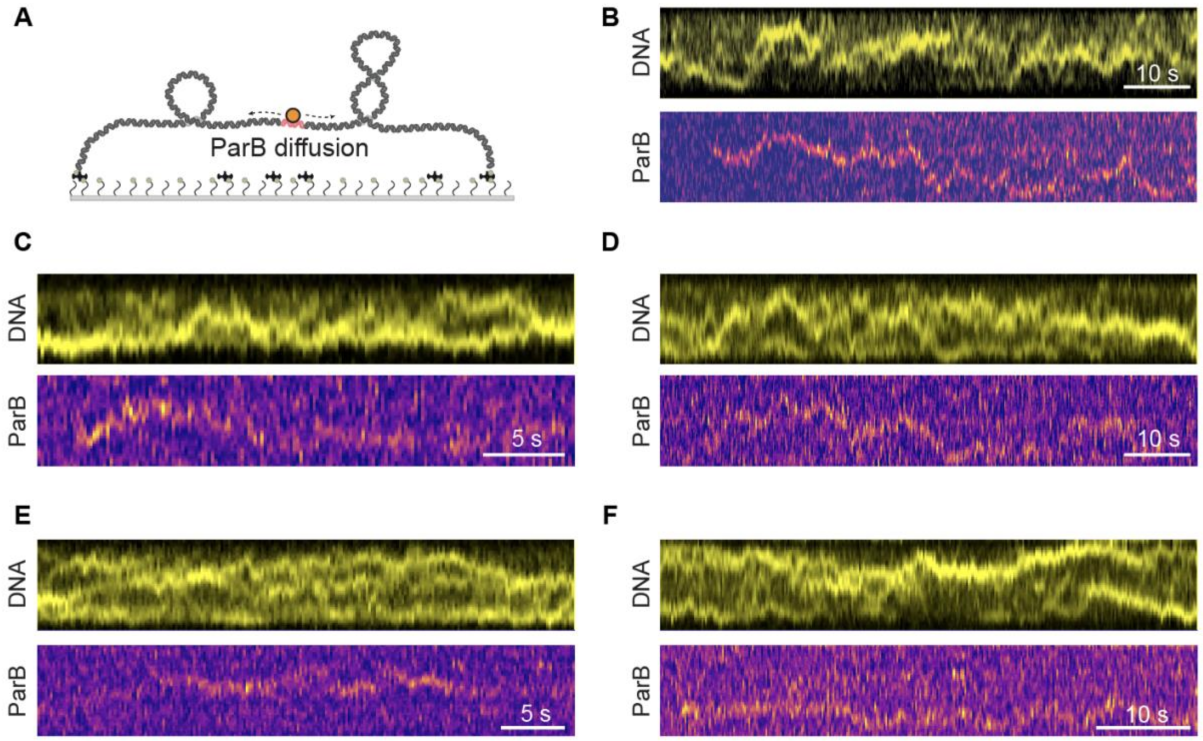

Figure S2. ParB diffusing along a supercoiled DNA after binding the *parS* sequence.
A) Schematic representation of the supercoiled DNA and diffusion of ParB proteins. **B-F)** Kymographs showing one-dimensional diffusion of a single ParB^{Alexa647} dimers (bottom) and DNA plectonemes (top) without strong plectoneme pinning.

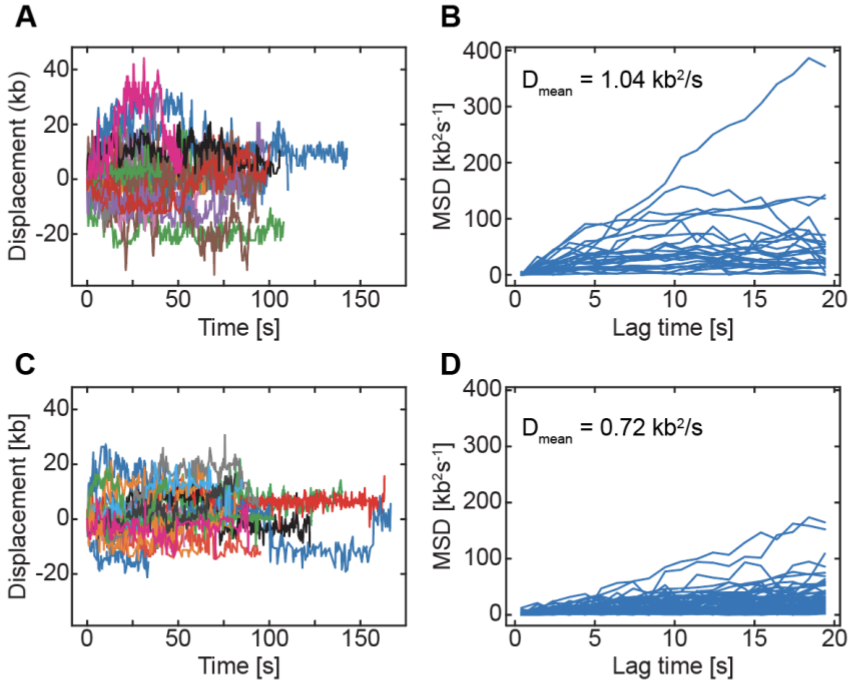

Figure S3. ParB exhibits faster diffusion on positively supercoiled DNA.

A) Single ParB diffusion traces along the positively supercoiled 38 kb DNA molecule. **B)** Mean squared displacement (MSD) of individual ParB traces. D_{mean} = 1.04 ± 0.26 kb^2/s (mean ± SEM), D_{median} = 0.69 ± 0.35 kb^2/s (median ± SE), n = 66. **C-D)** Same as A-B but for non-coilable DNA molecules. **D)** D_{mean} = 0.72 ± 0.11 kb^2/s (mean ± SEM), D_{median} = 0.43 ± 0.13 kb^2/s (median ± SE), n = 58.

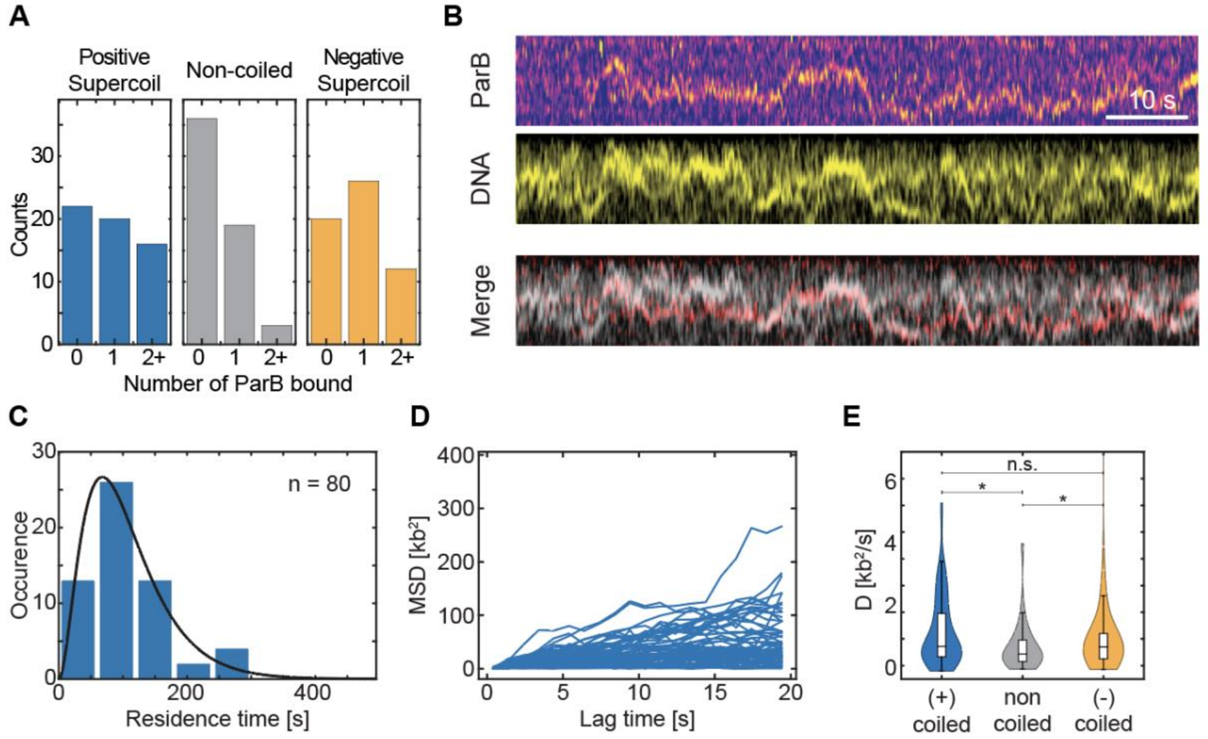

Figure S4. ParB efficiently binds and diffuses along negatively supercoiled DNA. A) Quantitative analysis of ParB molecules binding to positively coiled (Left), non-coiled (Middle) and negatively coiled (Right) DNA along 2000 frames at concentration of 0.1 nM. **B)** Kymographs showing one-dimensional diffusion of a single $ParB^{Alexa647}$ dimers (top) and DNA plectonemes (middle). **C)** Residence times of diffusing ParB dimers after binding to the *parS* site. The data were fitted to a model assuming a delayed dissociation of ParB from the DNA after CTP hydrolysis of both nucleotides (black line, see Tišma et al. 2022). **D)** Mean squared displacement (MSD) of individual ParB traces on negatively supercoiled DNA. $D_{mean} = 1.31 \pm 0.20$ kb^2/s (mean ± std), $D_{median} = 0.59 \pm 0.25$ kb^2/s (median ± SE), n = 57. **E)** Diffusion coefficient of ParB molecules on non-coiled, negatively coiled and positively coiled DNA molecules (N: 58; 76; 70). P-values: p(negative-positive) = 0.14; p(negative-noncoiled) = 0.033; p(positive-noncoiled) = 0.014.

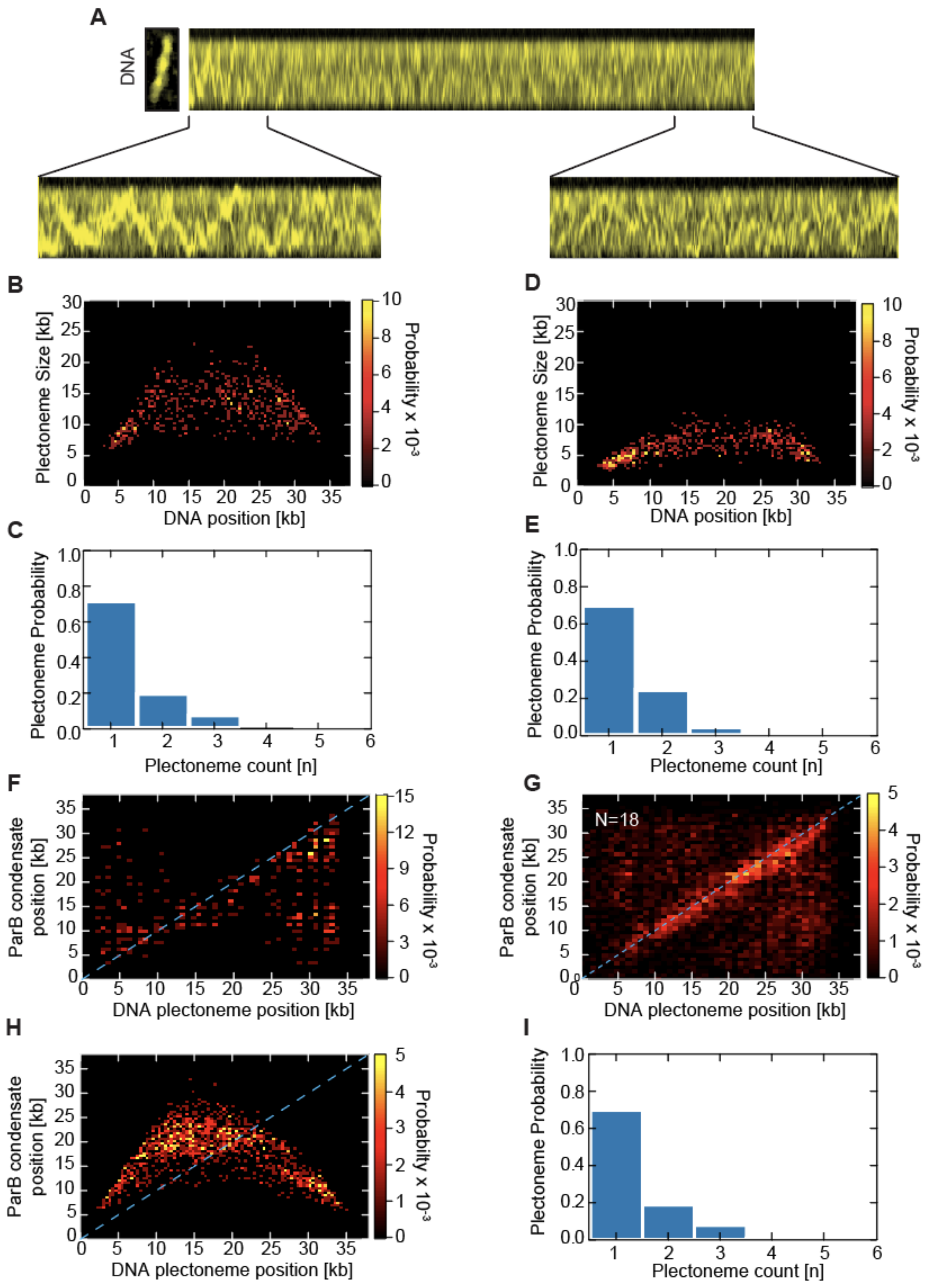

Figure S5. ParB proteins at low concentration do not pin DNA plectonemes. A) Kymographs showing supercoiled DNA at 3 nM [ParB] in the single-molecule assay. **B)** Probability distribution of plectoneme size versus DNA position before protein binding at 3 nM ParB protein. **C)** Observed number of co-existing plectonemes on the supercoiled DNA before the presence of ParB. **D)** Probability distribution of plectoneme size versus DNA position after protein binding at 3 nM ParB protein. **E)** Observed number of co-existing plectonemes on the supercoiled DNA in the presence of ParB. **F)** Heatmap showing the spatial correlation between ParB brightest condensate and brightest DNA plectoneme. **G)** Same as G) but cumulative for all molecules. **H)** Probability distribution of plectoneme size versus DNA position before protein binding at 0 nM ParB protein as a control. **I)** Observed number of co-existing plectonemes on the supercoiled DNA in the absence of ParB (show in G).

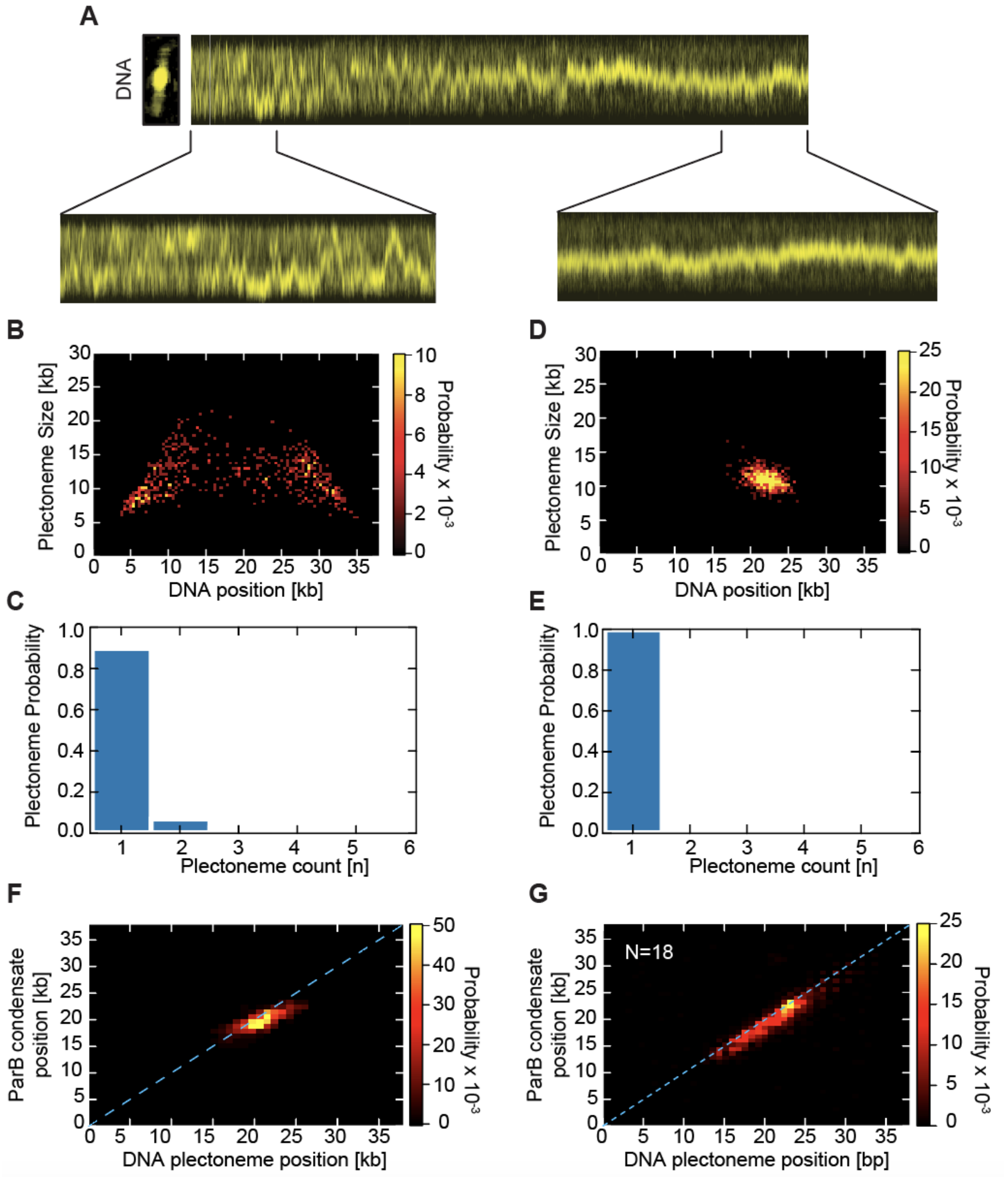

Figure S6. Multiple ParB proteins pin the plectonemes into a single static cluster at high concentrations. A) Kymographs showing supercoiled DNA at 25 nM [ParB] in the single-molecule assay. **B)** Probability distribution of plectoneme size versus DNA position before protein binding of ParB protein. **C)** Observed number of co-existing plectonemes on the supercoiled DNA before the presence of ParB (related to panel B). **D)** Probability distribution of plectoneme size versus DNA position after ParB protein binding at 25 nM concentration. **E)** Observed number of co-existing plectonemes on the supercoiled DNA in the presence of ParB (related to D). **F)** Spatial correlation between ParB brightest condensate and brightest DNA plectoneme. Blue line indicates the line of perfect correlation (r = 1). **G)** Same as in panel F but cumulative for all molecules (N=18).

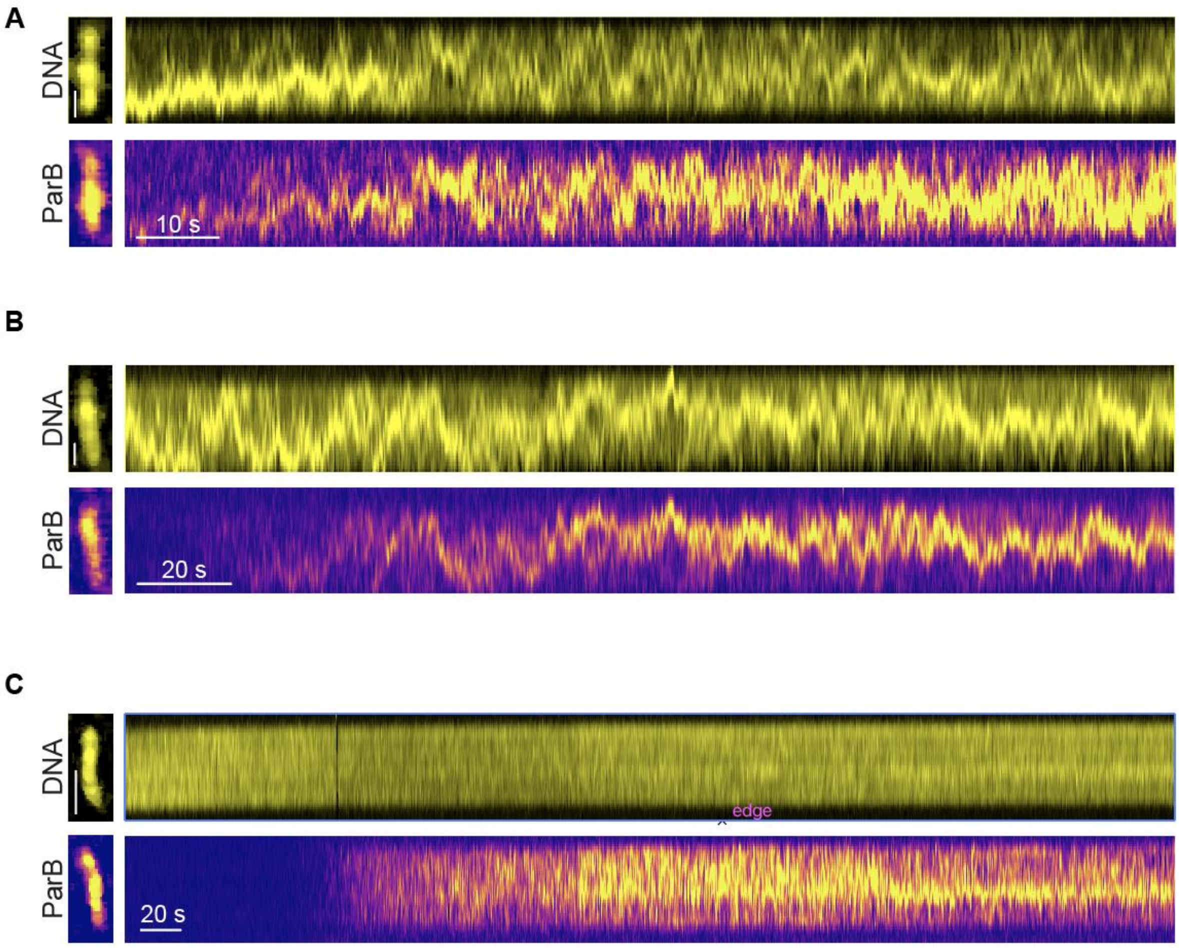

Figure S7. ParB spreading is not affected by DNA supercoiling.

A-C) Kymographs of ParB spreading over the entirety of the 38 kb DNA molecule at the concentration of 25nM. **A)** ParB spreading on positively supercoiled DNA. Spatial scale bar = 1 µm **B)** ParB spreading on negatively supercoiled DNA. Spatial scale bar = 1 µm. **C)** ParB spreading on non-coiled DNA molecule. Spatial scale bar = 2 µm.

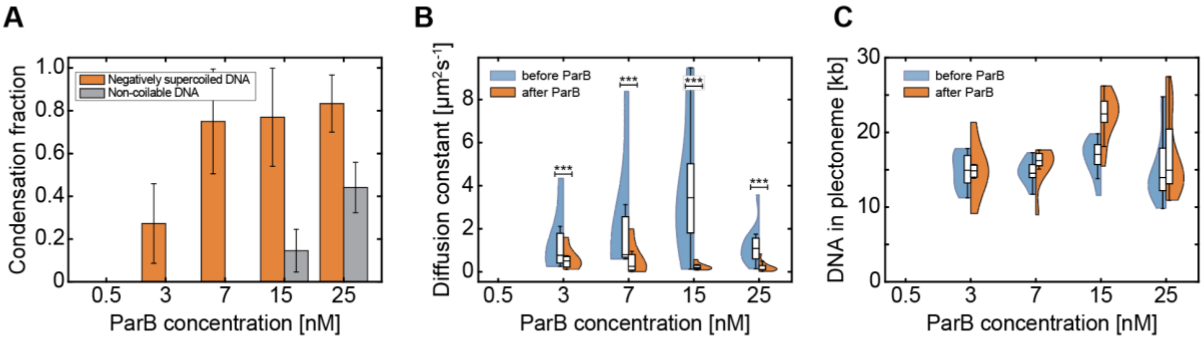

Figure S8. DNA-condensation by ParB proteins is facilitated by DNA negative-supercoiling.

A) Fraction of condensed DNA molecules in the presence of an increasing concentration of ParB proteins on non-coiled (orange) and negatively supercoiled DNA (blue). (N - 0.5nM:16; 3nM:22; 7nM:18; 15nM:17; 25nM:30) **B)** Dynamics of DNA plectonemes/condensed plectonemes in the presence of increasing concentration of ParB proteins. Blue – before ParB enters the flow channel. Orange – after >10 minutes after ParB was added to the flow channel and is covering the DNA_{parS} molecules (p-values: 0.5nM:N/A; 3nM:0.93; 7nM:0.31; 15nM:<0.001; 25nM:<0.001). **C)** Total amount of DNA in plectonemes or in a negatively supercoiled condensate on the 38 kb DNA_{parS} molecules before (blue) and after (orange) the addition of ParB at shown concentration.

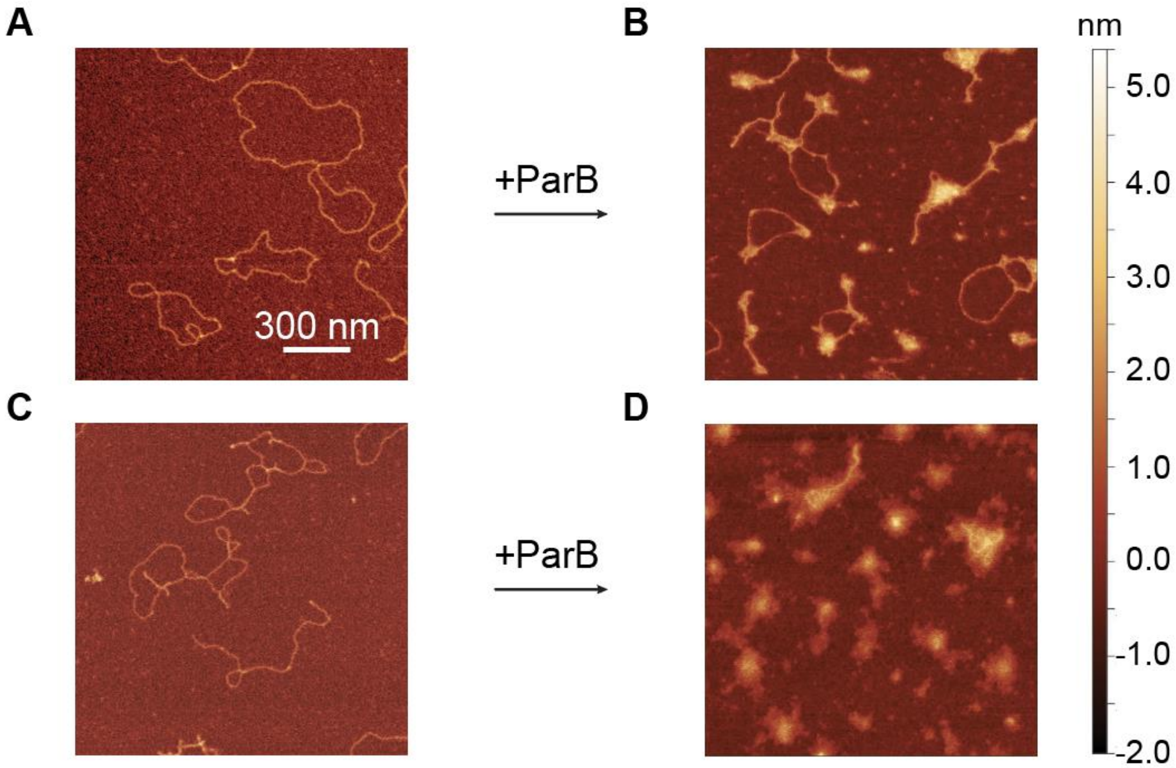

Figure S9. ParB proteins collapse the extended structures of DNA plectonemes.

A) AFM image of a nicked DNA plasmid with a length of 4.2 kb. **B)** AFM image of a nicked DNA incubated with 15 nM of ParB for 5 minutes leading to the formation of small DNA-condensates. **C)** Same as A but the plasmid was maintained in a supercoiled state. **D)** AFM image of a supercoiled DNA molecule incubated with 15 nM of ParB for 5 minutes leading to the collapse of extended DNA plectonemes into condensed structures.
