# Supplementary material for: DNA supercoiling enhances DNA condensation by ParB proteins
Source: Nucleic Acids Res. 2024 Oct 23;52(21):13255–68. doi: 10.1093/nar/gkae936 (PMC11602141; doi:10.1093/nar/gkae936)
Supplement: gkae936_Supplemental_Files [file gkae936_supplemental_files.zip › Martin-Gonzalez et al_Supp_Mov_Legend.pdf]

Movie S1. ***In vitro*** **single-molecule dynamics of supercoiled DNA containing DNA plectonemes**
